# Predictive Dynamics of Human Pain Perception

**DOI:** 10.1371/journal.pcbi.1002719

**Published:** 2012-10-25

**Authors:** Guillermo A. Cecchi, Lejian Huang, Javeria Ali Hashmi, Marwan Baliki, María V. Centeno, Irina Rish, A. Vania Apkarian

**Affiliations:** 1 Computational Biology Center, T.J. Watson IBM Research Laboratory, Yorktown Heights, New York, United States of America; 2 Department of Physiology, Feinberg School of Medicine, Northwestern University, Chicago, Illinois, United States of America; Research Center Jülich, Germany

## Abstract

While the static magnitude of thermal pain perception has been shown to follow a power-law function of the temperature, its dynamical features have been largely overlooked. Due to the slow temporal experience of pain, multiple studies now show that the time evolution of its magnitude can be captured with continuous online ratings. Here we use such ratings to model quantitatively the temporal dynamics of thermal pain perception. We show that a differential equation captures the details of the temporal evolution in pain ratings in individual subjects for different stimulus pattern complexities, and also demonstrates strong predictive power to infer pain ratings, including readouts based only on brain functional images.

## Author Summary

We propose a model of thermal pain perception that accounts for its dynamical behavior, and can be used to predict subjective responses to thermal stimulation on individual subjects with high accuracy, close to 90% averaged over subjects (over 65% for the null hypothesis). The model is based on behavioral considerations that include the need to signal current or approaching tissue damage, and the need to discount past danger. Moreover, we show that in a ‘mind reading’ setting, the combined use of sparse regression to infer pain perception from functional MRI recordings (fMRI), and from the model applied to the stimulus temperature also inferred from fMRI, leads to equally significant predictive accuracy, close to 75% averaged over subjects. Our results demonstrate that a subjective percept such as pain displays a highly deterministic behavior.

## Introduction

Any scientific or philosophical examination of human perception invariably must take into consideration the long-lasting notion of the subjectivity of pain. Plato, Aristotle, Galen, and Darwin excluded pain from other sensory modalities and instead classified it with emotions. Avicenna (or Ibn Sina), the 11^th^ century Arab-Persian philosopher-physician, is credited to be the first to suggest pain as a specific skin sense; this idea was later reformulated by Descartes, who conceptualized pain signaling from the skin to the brain [1], [2]. The notion of subjectivity and thus incommunicability of personal pain was seminal in Wittgenstein's abandonment of logic and shifting the emphasis of 20th century philosophical inquiry towards the study of language, in order to understand how such a private experience can be communicated at all [3]. More recently, D. Dennett has argued, based on modern neuro-scientific understanding that due to its subjective nature, and in contrast to visual perception, pain cannot be captured in computational models [4]. Indeed, the official definition of pain as accepted by the International Association for the Study of Pain states that pain is “an unpleasant sensory and emotional experience”, and expands to assert that, “pain is always subjective” [5].

In contrast, psychophysics from its inception in the 19th century has attempted to demonstrate that at least parts of human experience/perception can be captured quantitatively and described with simple models. Beginning with the work of E.H. Weber and culminating with S.S. Stevens's law of magnitude perception, statistical properties of pain have been quantified and modeled using simple equations [6]–[8]. Currently, statistics of pain are most commonly quantified with questionnaire-based tools, and these remain the main instruments with which efficacy of pain therapies are studied in clinical trials, for example [9], [10]. Temporal profiles of pain perception, however, have been seldom studied [11]–[13]. Yet, with the advent of human brain imaging technology the need for tracking pain perception in time prompted a number of groups to study pain perception as a time-evolving phenomenon [14]–[17].

A result that has surprised the pain research community is the presence of strong temporal non-linearities in the relationship between the stimulus pattern and the corresponding ratings, including illusory perception of heat and warmth [16] which do not appear to fit any cogent framework and yet can be linked to brain activity [18], [19]. With this as a starting point, we treat here time evolution of acute thermal pain perception as a dynamical system described by differential equations, the properties of which provide a general summary of the transformation of thermal heat parameters to pain perception space. Surprisingly, simple and interpretable first- and second-order differential equations with very few parameters accurately model time variability of pain perception in humans elicited by thermal stimulation patterns of varying complexity. The equations can be used to infer with high accuracy the response of individuals in modeling conditions that include access to the stimulus temperature and in ‘mind reading’ setups, i.e. when pain perception is solely inferred from functional images of the brain aided by the derived equations.

## Results

### Psychophysics Modeling

Given that perception of pain is a slow event and can be rated continuously, online continuous ratings of thermal pain can be readily generated [14]–[17]. When the stimulus intensity on the skin is monitored together with the resultant ratings of pain, one can view this as a system identification problem where the input and output are continuous time varying variables.

We reason that behavioral and evolutionary constraints require thermal pain to display three basic features. First and foremost, it must signal the threat of tissue damage: this is obviously determined by the current value of the skin temperature. The signal magnitude must monotonically increase with the temperature, although not necessarily linearly (as in fact, tissue damage is not linear with temperature). Following standard psychophysical practice, we consider the perceived magnitude of pain to be a positive quantity, i.e. we exclude the possibility of negative pain. Secondly, this magnitude must anticipate the possibility of damage, sounding the alarm of an imminent threat given the recent history of temperature values, independently of the current temperature. This information can be partially captured by the rate of change of the skin temperature. Finally, given its powerful hold on behavior, the intensity of pain perception must quickly decay once the threat of damage disappears, so as not to interfere with ongoing mental states [20], [21]. Following these basic principles, we model pain perception as a dynamical system using a second-order differential equation:
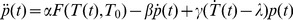
(1)Here 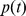 is the instantaneous perception of pain at time 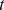, 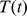 is the temperature, 
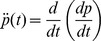
 is the pain acceleration, 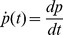 and 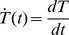 are, respectively, the pain's and temperature's rates of change. We explicitly constrain the dynamics to maintain the non-negativity of perception, 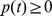, by imposing the boundary condition 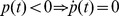.

**Figure 1:**
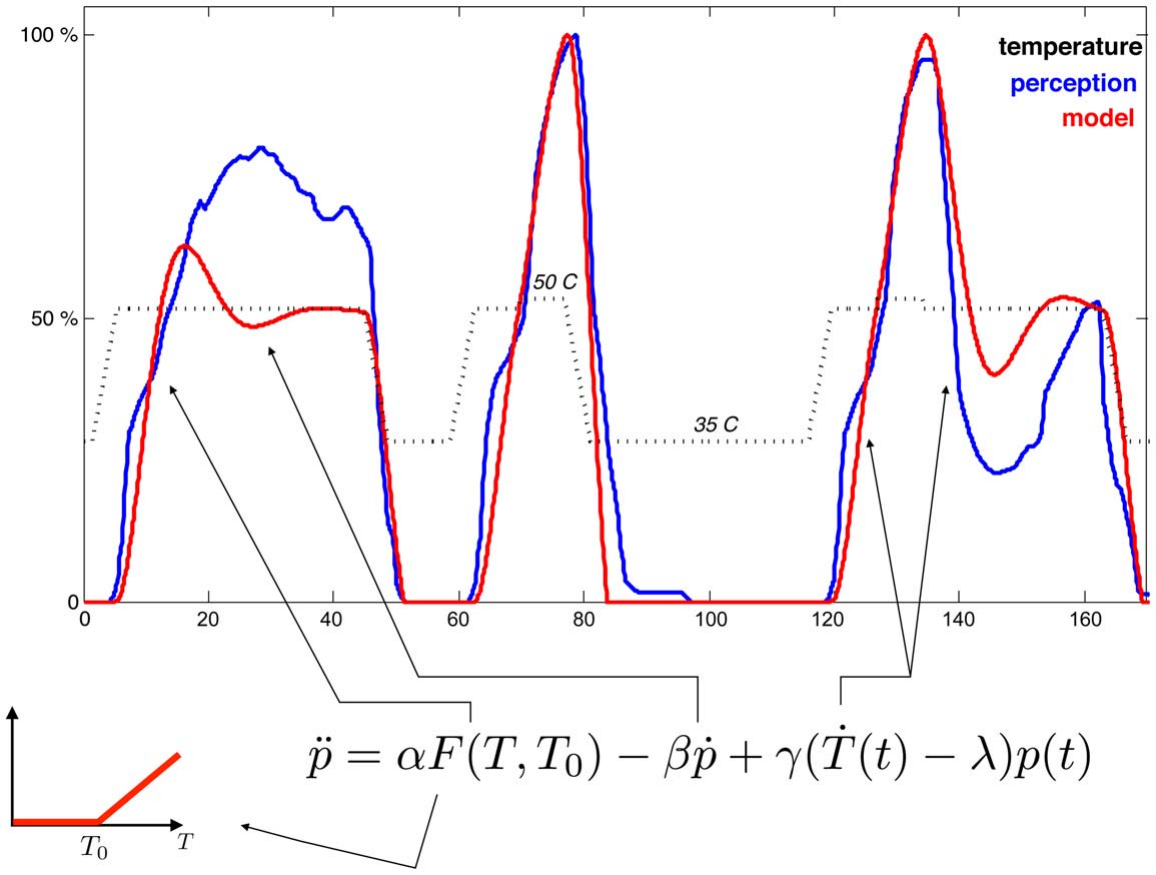
Reported perceptual pain level (blue), input temperature (dashed) and model (red). The experimental result is from [16] (their fig. 7). The vertical axis represents the subjective perceptual level (or applied temperature, dotted line), and the horizontal axis the time in seconds. The arrows point to the most evident effects of the three different components that drive pain perception: acceleration when the temperature exceeds the threshold (first arrow, left), dampening of oscillations (second arrow), and temperature change-dependent acceleration and restoring (third and fourth arrows). The small inset depicts the functional dependence of the forcing term with the temperature, which we chose to model as a ramp function. Time is in seconds.

The quantities 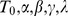 are subject-specific constants. The first term in the right-hand-side represents the temperature-dependent “force”, whose functional form we model, for the sake of parsimony, with a step function (**Figure 1** inset): 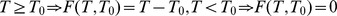, that is, the acceleration of the perception of pain takes effect only after the threshold 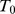 is exceeded. The second term is the decay of pain or “forgetting”, which helps perception return to its minimal value upon the removal of the injury threat presented by 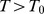, and also dampens the oscillations that naturally arise in a second-order dynamical system. The constant 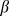 has units of 1/time, and therefore 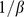 can be considered the time scale of the forgetting process. The third and last term is less intuitive, but equally meaningful from a functional perspective. It can be thought of as a dynamic restoring force, similar to the elastic term in the equation that describes a mechanical oscillator. When the derivative of the temperature is small enough, the term is negative and has the effect of limiting the pain level upon the continuing presence of a supra-threshold stimulus, as well as eliminating any sub-threshold pain fluctuations. When the temperature changes quickly, however, the effect of this term is more interesting. In the event of a temperature increase, the term becomes a driving force that helps accelerate the perception of pain, to build up an alerting signal that anticipates the upcoming threat of the temperature reaching and surpassing the injury threshold. Similarly, when the temperature drops fast, the term becomes a restoring force, pushing pain perception faster than the decay term and the passive restoring force would allow. Notice that this creates an asymmetry in the rise and fall time-constants, even when the rate of temperature change is the same in absolute terms: if the temperature drops when the pain perception is high, the restoration is much faster than the rise, for a similar rate of change of the temperature. The constant 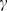 determines the intensity of the restoring/driving force, while 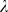 can be considered as a threshold above which fast changes in temperature become alarming.

The different effects of the three terms are illustrated in **Figure 1**, which depicts the evolution of pain perception averaged across subjects (blue trace) upon the presentation of an evolving temperature stimulus (dashed trace) (figure 7, from [16]; corresponding to our complex stimulus) and the best-fit inferred model (red trace). The temperature forcing term provides the basic effect of quickly increasing the magnitude of pain perception (first arrow on the left). An equilibrium intensity is reached by the combined limiting effects of the restoring force and the decay term (second arrow). The active form of restoring force (i.e. when 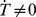) is most evident in the effect of the small kinks in temperature (third and fourth arrows).

In order to understand to what extent the complexity of the second-order dynamical system of Eq. 1 is warranted and the fit to the psychophysical pain ratings significant, we considered two null hypotheses and a model simplification to contrast our results. In the first place, we reasoned that the simplest approach for the nervous system to report thermal pain is by a direct correlation with the temperature, i.e. 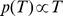. This null hypothesis is, in fact, too simple: the linear proportionality implies that temperatures a few degrees below the skin injury threshold will be reported only with proportionally weaker intensity than those a few degrees above the threshold. Alternatively, we considered a model in which perception is linearly proportional to the temperature, but only once it has exceeded a subject-dependent threshold. For obvious reasons, we termed these two null hypotheses as the linear and threshold-linear models, respectively; in the latter case, the temperature threshold is estimated by optimizing the correlation between model and data. The linear null hypothesis has several disadvantages; most glaring among them is the fact that it reports sub-threshold temperatures, which do not necessarily pose a threat of injury, almost as intensely as those that do pose a threat. Similarly, the threshold-linear model is impervious to events that fall below threshold but may signal an imminent threat, such as a sudden increase in temperature. To further probe the significance of our model, therefore, we considered a simpler first-order system derived from Eq. 1, assuming that the following conditions are satisfied: (a) the decay constant is sufficiently large, 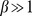 (i.e. the time scale 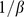 is short), and (b) the effect of the rate of change of the temperature is not significant, 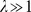. Simple algebra leads then to the following first-order differential equation:
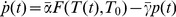
(2)Where 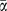 and 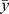 are subject-specific constants. The functional form of this equation is similar to that of a leaky capacitor, with the forcing affecting now the rate of change of perception (as opposed to the acceleration), and a restoring force that determines a unique time-constant 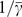 for both rising and falling of perception.

**Figure 2:**
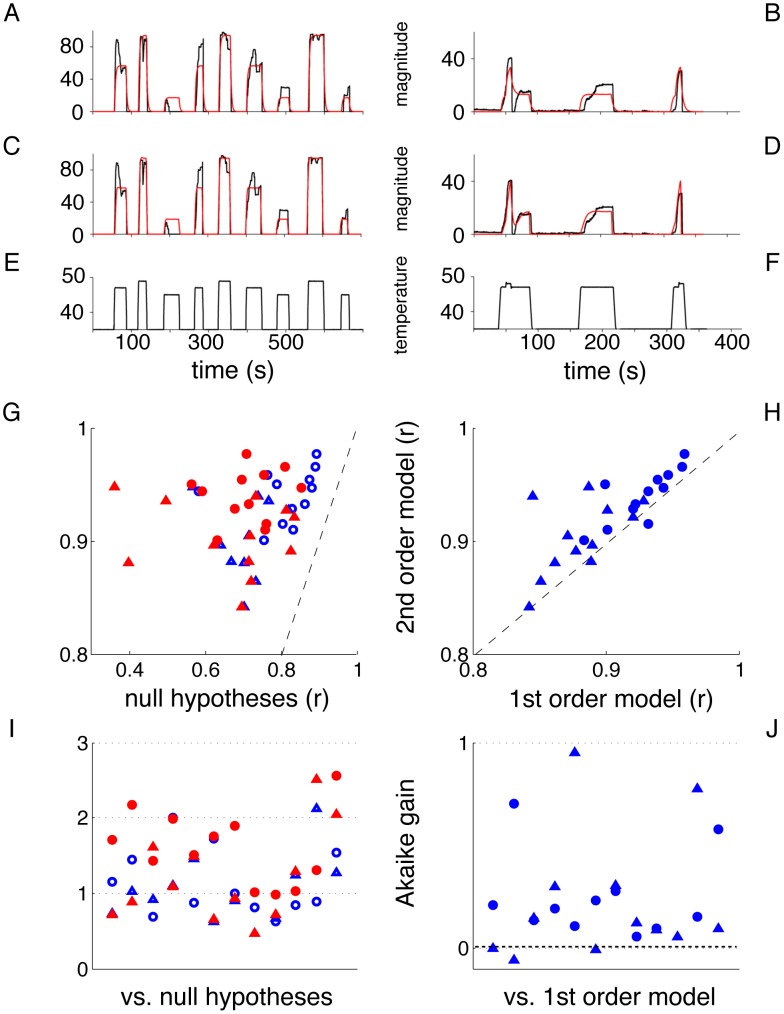
Examples of reported and modeled perception in a single subject, and adequacy of models across subjects. **A–F**: A single subject's pain rating (black) and fitted models (red), for a simple stimulus (E) using first (A) and second order (C) models, and for a complex stimulus (F) fitted by first (B) and second order (D) models. **G–H**: Performance comparisons between models. G: Comparing second order model to linear models, for simple (circle) and complex (triangle) stimuli. The linear model (red) corresponds to the null hypothesis that perception follows temperature, while the threshold model (blue) assumes perception follows temperature above a threshold with the temperature threshold optimized for each subject. H: Comparing first and second order models. Second order model outperforms linear and first order models; circles (triangles) correspond to simple (complex) stimuli. Correlation fit, r (zero lag Pearson correlation), measures accuracy of models to pain perception ratings. Each symbol/category is outcome for an individual subject. Dashed lines are the identity. **I–J**: Akaike Information Criterion analysis for the fits shown in panels G–H. The vertical axis corresponds to the gain in fit accuracy of the second order model over the null hypotheses (I) and the first order model (J), when a penalty for increased number of parameters is discounted. The color/shape is the same as for panels G–H; the horizontal axis corresponds to different subjects.

To test the relative merits of these models we performed psychophysical experiments, and contrasted model predictions. We designed two stimulation types: a simple stimulus in which the temperature ranges between a sub-threshold value and a supra-threshold value that is maintained constant during blocks [14], and a complex stimulus in which the blocks of supra-threshold temperature are interspersed with shorter blocks of higher temperature values [16] (see Methods for details). **Figure 2A–F** depicts an example of fitting a single subject's rating of a simple and a complex stimulus. Simple (panel E) and complex stimuli (panel F) are modeled using the first-order (panels A and B, respectively) and second-order models (panels C and D). Observe that while for the simple stimulus the two models appear to fit similarly well, the complex stimulus highlights the ability of the second-order model to capture the subtleties of the rating. Similar results were seen in all subjects studied (**Figure S1**).

The results of fitting the second-order model to the perceptual data for all participants are summarized in **Figure 2G**, showing the fit correlation for the second-order model contrasted with the null hypotheses. The increase of model performance over the null hypotheses is quite significant, reaching in some cases nearly 0.4, while the mean model correlation is above 0.9 (Wilcoxon matched-pairs signed-ranks test, Wp, 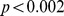). Similarly, the comparison with the first-order model (**Figure 2H**) shows that in all but two cases the second-order model is a better fit to the actual pain ratings (Wp, 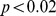). This increase in accuracy, however, may be explained by the model's larger number of parameters (5) compared with those for the simpler first-order model (3), and the two null hypotheses (1 for linear-threshold, none for linear). To account for this, we computed the difference in the Akaike Information Criterion (AIC) between the model and the null hypotheses. AIC regularizes the goodness of fit with a penalty for the number of free parameters in the model; **Figures 2I–J** show the gain in AIC for the model over the null hypotheses, and the first-order model, respectively, suggesting that overfitting can be ruled out (see Methods). To further assess our approach, we also compared the correlation between the derivatives of the rating and of the model (**Figure S2**), and again we observe that the second-order model outperforms the null hypotheses models (Wp, 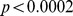) but not the first-order model (Wp, 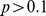).

We also considered the robustness and generalization capability of the modeling approach with respect to other sources of variability in the perceptual response. For that, we resorted to the concept of predictive modeling, a statistical learning approach that has gained increased acceptance in neuroscientific data analysis [22]: the parameters of a model are learned using training data, and then the goodness-of-fit evaluated on previously unutilized test data, as a means to estimate the model's generalization ability. We therefore computed the model parameters for each subject in the first run of the experiment, and estimated the response for the second, independent run using the same parameters. The results show that test and train correlations are still very similar (**Figures S6, S7A**). To understand the population effect of the stimulation paradigm and the modeling, we also fitted an average model of all the subjects, and then tested generalization efficacy of this model (**Figure S7B**). While the simple stimulus condition is not significantly affected, the complex stimulus shows a large decrement in the generalization ability of the model, indicating that responses to higher temporal structure are dependent on individual sensitivity parameters. A more rigorous test of generalization, however, involves predicting one class of stimuli in one run (i.e. complex in run 2) with parameters fitted to the other class and the other run (i.e. simple in run 1). Prediction of complex stimuli with parameters fitted to simple stimuli yields a group average of *r* = 0.68, over *r* = 0.93 for the estimate. Prediction of simple for parameters fitted to complex yields *r* = 0.84, very similar to the average of *r* = 0.89 for the estimate (see **Figure S7D–E**). The higher efficacy of the latter setup is consistent with the idea that the more complex stimuli can reveal the full dynamical structure of the responses, and therefore be more robust to generalization.

### Psychophysics and Physiology

**Figure 3:**
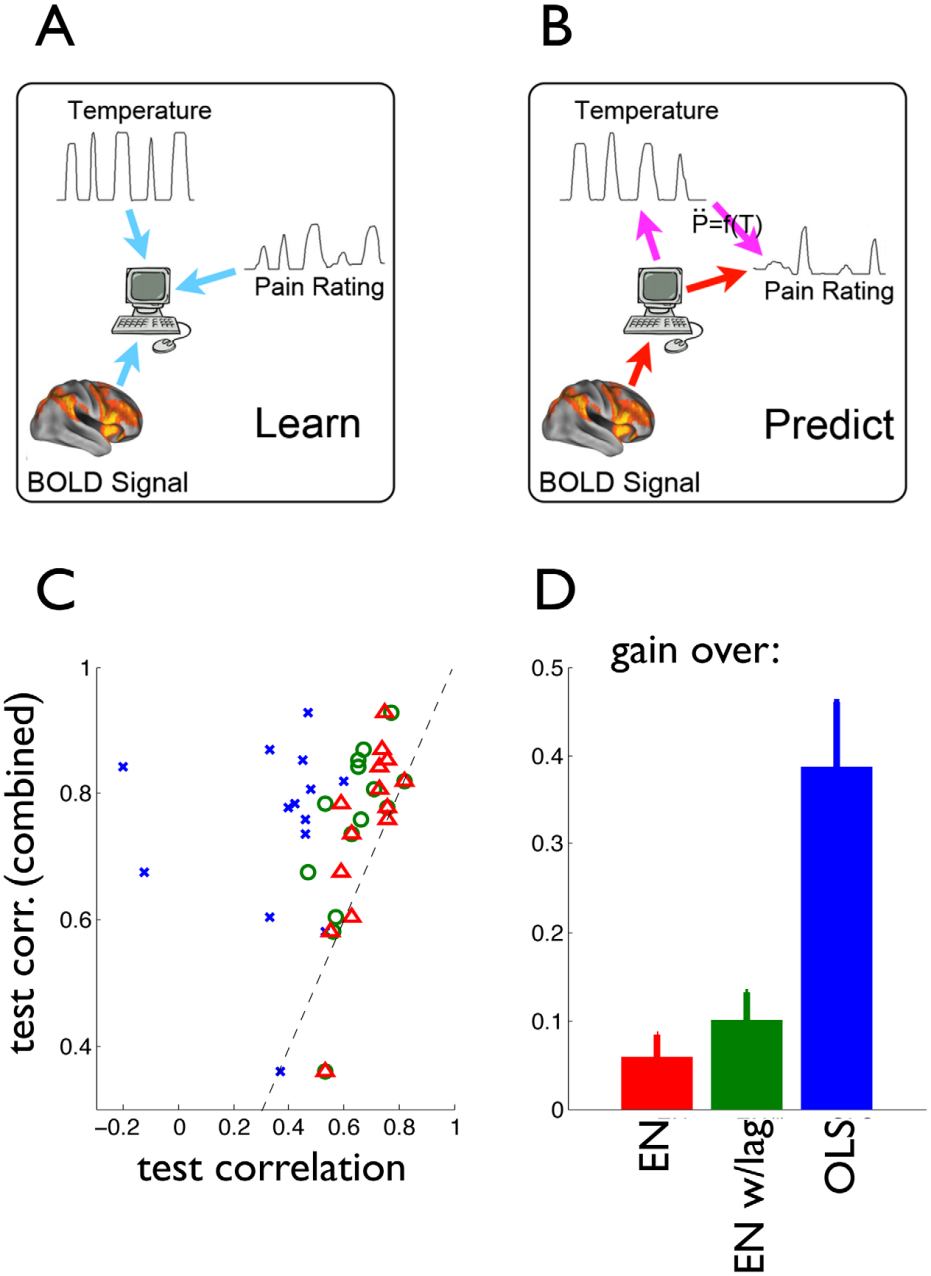
Scheme and performance for predictive modeling of pain ratings from brain activity. **A.** Depicts the learning phase, where the relationships between BOLD signal and temperature, BOLD signal and perception, and temperature and perception are learned on the training data. **B.** Corresponds to the prediction phase, where for three approaches (OLS, EN and EN w/lags) the learned model is used to infer the pain rating from the BOLD signal (red pathway), whereas for the Combined model the first inference is combined with the inference of temperature from BOLD, and perception from temperature using the second order model (magenta pathway). **C**: Predictive accuracy on test data of models inferring pain perception ratings from brain activity. The horizontal axis represents fit correlation between predicted and actual ratings for the three inference models that do not include the analytic dynamical model (OLS, EN and EN w/lags). The vertical axis is the predictive accuracy for the Combined model. Note the marked increase in prediction accuracy for the combined model. Each symbol/category is outcome for an individual subject. Dashed line is identity. **D**. Average gain in test correlation of the combined model over the three alternative predictive models.

One of the practical applications of predictive modeling in neuroscience is its use in “mind reading” setups, i.e. the possibility of obtaining precise information about perceptual and cognitive states, such as words or images presented to subjects in the fMRI scanner, by applying a predictive model to fMRI data [22]. The ability to predict and reconstruct with high accuracy external stimuli under certain conditions has proved to have enormous implications for basic research and brain-machine applications [23]–[25]; however, predictive modeling of clinically relevant measures has shown to be more elusive. To further demonstrate the relevance of our findings, we analyzed the impact of including the analytic model in a predictive setup, as follows: (**a**) we trained a predictive linear model with regularizing constraints, the Elastic Net [26], [27], to infer pain ratings from full-brain fMRI traces, utilizing TR volumes (i.e. the brain images acquired at each time point) concurrent with the ratings as independent samples (hereby labeled EN model); (**b**) we trained a model as in (a), but using up to 7 TR volumes previous to the time the ratings are reported, and using as predictors only voxels that have a time-lagged correlation with the target variable above a threshold (0.2 in this case) (EN w/lags model); (**c**) we trained a model as in (a) and combined it linearly with the analytic second order model, Eq. 1, trained on the same data using both temperature and pain ratings (Combined model). Specifically, the model is trained to infer the pain ratings from fMRI traces, independently infer the temperature from fMRI traces, obtain a second estimate of the pain ratings through the application of the dynamical model to the inferred temperature, and then combine both predictions into one. Finally, (**d**) we trained an unconstrained, linear ordinary least-squares model (OLS), with the same conditions as in (a) (**Figure 3A**).

With this setup, we then computed the predictive accuracy of the combined model to infer the pain ratings on unseen test data, using only the fMRI traces, and compared it with the predictions of the EN model, the EN w/lags model (to compensate for the intrinsic use of the recent history in the analytic model), and the OLS model (**Figure 3B**). The results are shown in **Figure 3C–D**, which displays for each subject the predictive accuracy of the EN, EN w/lags and OLS models in comparison to the Combined model. The Combined model shows a significant improvement in predictive accuracy over the other three models, including EN w/lags, which includes delayed information and helps it to predict better than EN. In all cases, the increase in accuracy is statistically significant (Wp, 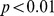). These results demonstrate that our dynamical model can be successfully combined with physiological measurements in order to obtain further insights into the mechanisms of pain perception, and eventually used as a scaffold for experimental manipulations. Moreover, given the high accuracy of the predictions, we conclude that “mind reading” of subjective pain perception is practically attainable.

### Model Consistency

Besides the model's predictive efficacy, it is important to understand how consistent it is with respect to the known phenomenology. In particular, the distribution of threshold temperatures over the population (**Figures S1, S4, S5**) closely matches classic values determined by rigorous psychophysical methods [28]. The other easily interpretable parameter of the model, the decay time-constant, also shows a reasonable distribution of values, as well as a good match between the second-order and the simplified first-order models (**Figures S1, S4, S5**).

In order to assess the significance of each of the terms contributing to the description of the perceptual dynamics in Eq. 1 and Eq. 2, we computed all pair-wise correlations between the corresponding fitted parameters in the second-order model. High correlation between two terms may indicate a redundancy in model, or perhaps an even worse inadequacy of the model to capture the essential features of the dynamics. Of all pairs (Table S1), only two reach statistical significance: between 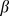 and 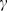
(*r* = 0.56, *p* = 0.01), and between 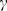
and 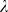 (*r* = −0.53, *p* = 0.017). It is instructive to contrast these values with the result of performing a similar computation with the fitted parameters for the first-order model; in this case, the correlation between 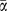 and 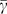 is significant (*r* = 0.72, *p* = 0.0003). A parsimonious interpretation of these results is that the simplification of the dynamics introduces correlations between terms that do not properly describe it. Given that the second-order model performs better, we conclude that the more complex model is also a better representation of the dynamics. Moreover, while the two correlations are significant, their actual value (*r* 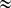
0.5) implies that their contributions are not redundant.

We tested more radical variants of the modeling approach, in order to test its goodness-of-fit in a “functional space”. In particular, Eq. 2 was expanded to incorporate two time-constants, slow and fast systems corresponding to the physiology of slow (unmyelinated) and fast conducting (myelinated) nociceptive afferents [29]; we determined that such models do not substantially improve prediction of pain ratings (**Figure S9**). In fact, the apparent presence of two time-constants in the perceptual dynamics is accounted for, in Eq. 1, by the 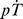 term, which models the decay of perception after the temperature drops below threshold as faster than the rising time-constant (because 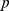 is higher in the former than in the latter, see **Figure 1**).

A large psychophysical body of literature shows that static ratings of thermal pain, similarly to other sensory modalities, follow S.S. Stevens's power-law for perceived magnitudes [8], suggesting that the dependence of dynamics of pain perception on temperature might be better modeled by a power function. As this law describes the stationary or steady-state response to pain, as opposed to its dynamical behavior, we cannot directly compare it against our model. However, we considered that it would be possible to extend the model to encompass power-law stationary responses. Given that this requires an additional parameter (the exponent), it is more reasonable to consider an extension of Eq. 2, in which the term driven by the difference between the current temperature and the threshold is modified by an exponent, leading to:
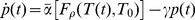
(3)where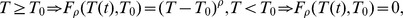 and 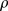 is an additional parameter. Performance of this new model was contrasted to Eq. 2, yielding results that are comparable but slightly poorer, even though the model has one more parameter. To summarize, the mean correlation over simple and complex stimuli was 0.90 and 0.87, compared to 0.92 and 0.88 for Eq. 2. We also observe that as long as 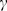 and 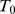 are fitted for individual ratings, proportionality constant 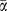 and the power parameter 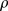 compensate for each other (range for 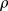 was 2.97 to −0.28, mean = 1.0 and SEM = 0.3), and 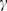 and 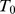 converge to the same optimal values as found for Eq. 2 (performance measure between Eq. 2 and Eq. 3 using either *r* or *SSE* shows a correlation of 0.99, *p* = 0).

Our model can capture, in a single framework, perceptual behaviors that are usually considered as disparate. Given that the perception of pain can be parceled into separate dimensions and as recent evidence suggests that the temporal dynamics of these modalities may have unique properties that depend on stimulus intensity [28], we examined the properties of our models for the percept of burning. When subjects were instructed to report the magnitude of burning pain [28], we observed similar rating profiles and model fitting to the perceived magnitude of pain, indicating that the modeling approach may be equally applicable to sub-modalities of pain.

Similarly, our model encompasses the different behaviors associated with offset analgesia (OA). While OA is usually defined by the de-sensitization to the same noxious temperature following exposure to a more noxious one [16] (a feature essentially captured by our model, cfr. **Figure 1**), other more subtle features have been reported in the literature under the OA characterization, of which we will consider the main two. The first one is the observation that temperature fall rates in the range of 0.1 to 0.5°C/sec are barely detected with continuous ratings of pain [16]. We tested whether our second order model will also show less sensitivity to stimulus offset rates, in comparison to the first order model, where perception fall rates should better reflect stimulus fall rates. **Figure S10** shows that in fact these predictions are correct (the model closely captures pain ratings as described in figures 3 and 4 in [16]). A second observation regarding OA is that pain perception magnitude for increasing intensities shows different patterns when the stimulus has an additional one degree perturbation (offset stimulus) in contrast to when the stimulus is kept at a constant level or returns to baseline [30]. Again our second order model captures these features better than the first order (**Figure S11**), and in fact our model replicates figures 2–5 in [30].

## Methods

### Psychophysics

#### Participants

Twelve healthy subjects participated in this part of the study: 6 women and 6 men (Age: 26±0.3 years; mean ± S.D.). All subjects were right-handed, and all gave informed consent to procedures approved by Northwestern University IRB committee.

#### Thermal stimuli and psychophysical ratings

Stimuli were delivered to the dorsal aspect of the right arm with a thermal stimulator (3×3 cm Peltier) (Medoc TSA-2001; Israel). Two types of stimulus series, simple and complex, were applied in a randomized order at different skin locations. The simple stimulus started at baseline 35°C, with peak temperatures 45°C, 47°C and 49°C, nine stimuli ranging in duration from 10 to 40 s. Durations, intensities, and inter-stimulus intervals were pseudo-randomized. The complex stimuli consisted of three stimulus pulses adapted from [16]: from baseline 35°C sustained for 30 sec the initial peak was 47°C, after 5 sec the skin temperature further increased by 1°C sustained for 5 sec, then returned to 47°C for 20 sec. After a 50 sec baseline adaptation, the second stimulus pulse was applied at 47°C for 35 sec. This was followed by 60 sec baseline adaptation and third pulse consisting of a 47°C, 5 sec stimulus followed by a 48°C, 5 sec stimulus. Stimulus rise and fall rates were about 8°C/s (**see** **Figure 2**, and **Figure S1**).

Subjects continuously rated the perceived pain intensity for simple and complex stimuli using a finger-span device. The anchors were “no pain” at the lower limit of 0 and “most intense pain imaginable” at 100. The finger span device was comprised of a potentiometer the voltage of which was digitized and connected to a computer providing visual feedback. Participants underwent an initial training phase prior to data collection. Every subject performed pain intensity rating for simple and complex stimuli twice in a randomized order. These subjects were also asked to rate only the intensity of dull burning sensation evoked by the simple or complex stimulus in two additional runs, presented in a randomised order and interspersed between the pain intensity rating runs.

### Model Simulation and Parameter Estimation

Model simulation was implemented with standard integration algorithms in Matlab. To obtain the simplified Eq. 2 from Eq. 1, we write
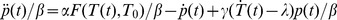
Assuming a large decay constant (equiv. a short time scale to ‘forget’), 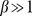 and that the effect of fast changes in the temperature profile is negligible, 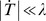, we can drop the l.h.s. term to write
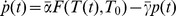
Where 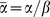 and 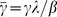.

Parameter estimates for first order and second order equations were calculated in Matlab using minimization of the least squares error between simulation and experimental data, and a random search technique over the parameter space. For each stimulus rating condition, three parameters were calculated for first order fitting and five parameters for second order fitting. Adequacy of fitting was measured by zero-lag Pearson correlation between model output and pain ratings.

Overfitting of the model was investigated using the Akaike Information Criterion (AIC), which penalizes the measure of goodness of fit with a term proportional to the number of free parameters [31]. When the residual squared error sum (*SS*) is known, the criterion can be written as
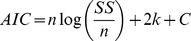
where *n* is the number of samples, and *k* the number of parameters. 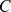 is a constant that depends on the particular dataset used, but not on the model, and therefore can be ignored when making comparisons of 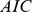 between models for the same data. As even when 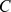 is discounted, this measure still depends on the total number of samples, for presentation's sake we computed a normalized version, which we call here the Akaike gain for the model (*m*) with respect to the contrasting null hypotheses and first-order model (*c*), as
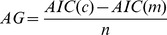
A positive value for 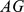 indicates that the gain in accuracy of the model cannot be explained by the increase in number of parameters. For the first null hypothesis, i.e. perception proportional to temperature, the number of parameters is zero. The second null hypothesis, perception proportional to temperature over a threshold, has one free parameter that we estimate similarly to the analytic models.

The Pearson correlation between the parameters for the second-order and first-order models was computed using all fitted parameters across subjects and stimuli (**Table S1**).

### fMRI

The functional MRI data are the same used in an earlier study [14]. Here the thermal stimulus and related ratings of pain are used to compare results of full-brain machine learning with elastic net for predicting pain perception with and without incorporation of our quantitative model for pain perception, Eq. 1.

#### Participants

Fourteen healthy subjects participated in the study: 7 women and 7 men (Age: 35.21±11.48 years; mean ± S.D.). All subjects were right-handed, and all gave informed consent to procedures approved by Northwestern University IRB committee.

#### Pain rating task

Subjects were scanned while rating their pain in response to thermal stimuli applied to their back (pain rating task) using a finger-span device. Participants underwent an initial training phase prior to scanning. The finger span device was comprised of a potentiometer the voltage of which was digitized and time-stamped in reference to fMRI image acquisition and connected to a computer providing visual feedback. A purpose built, fMRI compatible thermal stimulator delivered painful thermal stimuli, simple sequence in the psychophysics study, was applied to the lower back at midline twice, resulting in separate fMRI data sets.

#### fMRI data acquisition and preprocessing

Functional MR data was acquired with a 3T Siemens Trio whole-body scanner with echo-planar imaging capability using the standard radio-frequency head coil. Multi-slice T2*-weighted echo-planar images were obtained: repetition time TR = 2.5 sec, echo time TE = 30 msec, flip angle = 90°, slice thickness = 3 mm, in-plane resolution = 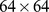. The 36 slices covered the whole brain from the cerebellum to the vertex. A T1-weighted anatomical MRI image was also acquired for each subject using the following parameters: TR = 2.1 s, TE = 4.38 ms, flip angle = 8°, FOV = 220 mm, slice thickness = 1 mm, in-plane resolution = 0.86×0.86 mm^2^ and number of sagittal slices = 160.

Image pre-processing prior to using fMRI in predicting pain perception based on changes in BOLD signal was performed on each subject's data using FMRIB Expert Analysis Tool (FEAT, www.fmrib.ox.ac.uk/fsl). The pre-processing of time-series of fMRI volumes encompassed: skull extraction using BET; slice time correction; motion correction; spatial smoothing using a Gaussian kernel of full-width-half-maximum 5 mm; non-linear high-pass temporal filtering (120 seconds) and subtraction of the mean of each voxel time-course from that time-course. Six time series obtained from rigid head motion corrections were used as covariates of no interest, to remove residual variance due to head motion.

### Machine Learning with Elastic Net (EN) to Predict Pain Perception from fMRI Activity

Herein, we learn a predictive model individually for each subject. We treat voxels as predictor variables, TRs as independent samples (following [26], [27]), and pain ratings as target variables, respectively. While the independence assumption among subsequent TRs does not hold in practice, and is used mainly for simplicity sake, it allows us to reach good predictive accuracy. We learn the model parameters using the first half of the experiment as training data, and then apply the model to the second half of the experiments, treated here as test data.

Sparse predictive models were learned using a sparse regression method called the Elastic Net [32], which enhances the basic LASSO regression [33] by combining <$>\raster(80%)="rg1"<$>*1*-norm (sparsity-enforcing) constraint with the <$>\raster(80%)="rg1"<$>2-norm (“grouping”) constraint. The rationale behind this extension is to overcome a known limitation of the LASSO: given groups of correlated variables (e.g., spatial clusters of voxels), LASSO may pick an arbitrary one from the group, as long as the resulting model predicts well; however, if the goal is neuro-scientific interpretation of the sparse model as a set of voxels relevant to the task, it is important to include (or exclude) voxels as groups (clusters) of highly-correlated variables, rather than single representatives of a group. This is achieved, to some extent, by controlling the grouping parameter mentioned above, that tends to enforce similar coefficients among highly correlated voxels (e.g., spatial neighbors). The Elastic Net and other models used in this paper are formally described below, and summarized in **Table 1**.

**Table 1:** Models summary.

	Data for Learning	Data for Prediction	Predicts
OLS	p(t)	fMRI(t)	p(t)
	fMRI(t)		
EN	p(t)	fMRI(t)	p(t)
	fMRI(t)		
EN w/lags	p(t)	fMRI(t,t-1,..,t-7)	p(t)
	fMRI(t,t-1,..,t-7)		
Combined analytic model + EN	p(t,t-1)	fMRI(t)	p(t)
	T(t,t-1)		T(t)
	fMRI(t)		

The table summarizes the four predictive models considered for the inference of pain perception from the fMRI signal. Note that the combined model implicitly predicts the temperature, but we have not considered it in the present article.

#### Ordinary Least Squares (OLS) model

Let *X_1_,…,X_n_* be a set of N predictor variables (predictors), such as voxel's intensities, or BOLD signals, and let *Y* be the response variable, such as a pain perception rating. Let ***X*** = (***x*** *_1_*|…|***x*** *_n_*) denote the *m*×*n* data matrix, where each **x** is an *m*-dimensional vector consisting of the values for predictor *X_i_* for *m* data samples, while the *m*-dimensional vector ***y*** denotes the corresponding values for the response variable *Y*. We consider the problem of estimating the coefficients *β_i_* in the following linear regression model:
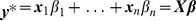
(4)where y* is an approximation of ***y***. As a baseline, we use the Ordinary Least Squares (OLS) regression approach which finds a set of *β_i_* that minimize the sum-squared approximation error:
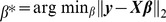
(5)Where ∥**.**∥*_2_* represents the <$>\raster(80%)="rg1"<$>*2*-norm. When **X** has the full column-rank (which also implies that the number of samples *m* is larger than the number of variables *n*), OLS finds the closed-form unique solution β* = inv (***X^T^ X***) ***X^T^ y***, where inv() denotes the matrix inverse, and ***X^T^*** denotes the matrix transpose, respectively. However, when *n>m*, as it is often the case in fMRI data with thousands of predictors (voxels) and only a few hundreds of samples (TRs), there is no unique solution to the OLS problem, and additional constraints are required to “regularize” the problem. Moreover, predictive accuracy of OLS solutions can be low due to over-fitting in high-dimensional, but small-sample problems. Finally, OLS does not perform any automatic variable selection (i.e., all coefficients tend to be nonzero), so that it is hard to identify which predictors (e.g., voxels) are most relevant to the response variable.

In the past decades, various regularization approaches have been proposed in order to improve OLS models to handle properly large-*n*, small-*m* datasets, and to avoid the over-fitting (e.g., ridge regression [34], bridge regression [35], LASSO regression [33], and so on. Specifically, recently proposed sparse regularization methods such as LASSO [33] and Elastic Net [32] address both of the OLS shortcomings, since variable selection is embedded into their model-fitting process. Sparse regularization methods include the <$>\raster(80%)="rg1"<$>1-norm penalty on the coefficients, which is known to produce sparse solutions, i.e. solutions with many zeros, thus eliminating predictors that are not essential.

#### Elastic net model

In this paper, we use the Elastic Net (EN) regression approach. This algorithm finds an optimal solution to the least-squares (OLS) problem, augmented with additional regularization terms that include the sparsity-enforcing <$>\raster(80%)="rg1"<$>1-norm constraint on the regression coefficients that “shrinks” some coefficients to zero, and a “grouping” <$>\raster(80%)="rg1"<$>2-norm constraint that enforces similar coefficients on predictors that are highly correlated with each other, thus allowing selection of relevant groups of voxels, which <$>\raster(80%)="rg1"<$>1-norm constraint alone is not providing. This can improve the interpretability of the model, for example, including a group of similarly relevant voxels, rather than one representative voxel from the group. Formally, EN regression optimizes the following function
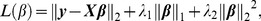
(6)where ∥**.**∥*_1_* and ∥**.**∥*_2_^2^* represent the <$>\raster(80%)="rg1"<$>*1*-norm and (squared) <$>\raster(80%)="rg1"<$>*2*-norm, respectively.

In order to solve the EN problem, we use the LARS-EN algorithm of [32]. It takes as an input the grouping parameter *λ_2_* and the sparsity parameter that explicitly specifies the desired number of selected predictors; this number corresponds to a unique value of *λ_1_* in Eq. 3. Thus, herein we will slightly abuse the notation, and following [26] denote the sparsity parameter as *λ_1_* while always interpreting it as the number of selected predictors.

#### EN w/lags model

When predicting a stimulus or behavior from fMRI data, it is typical to use as the predictors the voxels intensities at the current TR, and treat TRs as independent and identically distributed (i.i.d.) samples [26]. While this assumption can lead to over-estimates of accuracy under auto-correlated noise, temporal information from the past TRs may sometimes improve the predictive model, as we demonstrate, for example, in [27]. We considered as a set of predictors all voxels from the past 7 TRs, and the current TR. However, due to very high dimensionality of this set, we selected only a subset of those voxels that were correlated with the response variable above the given threshold (herein, we used 0.2). (Note that time-lagged voxel's time series were shifted forward by the appropriate lag in order to properly align it with the response time series).

#### Combined model

The combined model was constructed as follows. First, we used the training fMRI data and the actual temperature recording for the corresponding TRs in order to learn an Elastic Net regression model for predicting the temperature stimulus from fMRI. Next, given the training data for temperature and pain perception, we learned the parameters of the analytical model, second-order differential equation, Eq. 1. We then combine the prediction of the EN fMRI-to-temperature-to-pain and the EN fMRI-to-pain models, learning the parameters of the combined model similarly on the train data.

More explicitly, in the testing phase we assume that the temperature is not actually known (i.e., is a “hidden variable”), and apply the learned EN regression model to the test fMRI data in order to predict the temperature stimulus. We then apply the analytical model (Eq. 1) to predict the pain perception given the predicted temperature, and combine this prediction with the direct EN regression model for pain in order to obtain the final prediction.

#### Training and testing

The data from the pain-stimulus rating session were split into the training and test subsets: the data associated with the first 120 TRs were used for training the models, while the remaining 120 TRs were used for testing the predictive accuracy. The accuracy of the model was measured by the Pearson's correlation coefficient between the response variable (pain rating) and its prediction by the model.

## Discussion

### Model Efficacy

The results show that acute thermal pain perception applied to healthy skin follows simple quantitative deterministic patterns. The dynamic model is derived from a behaviorally relevant interpretation of pain perception as a warning signal that quickly reports immediate threat of injury (temperature above threshold), and approaching danger (rapid temperature increases), and can also as easily discount the threat once it goes away or it is expected to do so (temperature decreases). The model, using few parameters, can reproduce with high accuracy the dynamical transformation from stimulus to perception. Moreover, the model also has high predictive accuracy, and accounts for subjects' variability with simple and interpretable mechanisms.

The model provides a summary of a relatively complex behavior, whose physiological correlates and mechanisms can be directly investigated through pharmacological manipulation and the design of targeted stimulus conditions. Temporal processing is ubiquitous in sensory systems, including the somatosensory pathway [36], [37]. However, it is only in a few cases that spatio-temporal transformations can be functionally interpreted, beyond generic sharpening for enhanced localization [38], or information compression [39]. We do not consider, however, that the perceptual dynamics captured by our model can be reduced to peripheral processing. In fact, as previously reported [14], the BOLD response to a task similar to the one used in this report reveals a rich temporal structure across several cortical and sub-cortical areas compatible with the time scale of the perceptual ratings, such that the dynamics of pain perception may result from the emergent interaction of extensive networks. Moreover, given its ultimately non-linear nature, the model further predicts dynamical features of pain perception that may have unexpected behavioral relevance (see **Text S1**).

The utilization of our analytic model within the “mind reading” setup highlights its predictive efficacy, and provides an additional validation step. A further reason for using the combined model, besides simply inferring pain from fMRI, is to go beyond the limitation of simple linear inference models such as Elastic Net, while keeping the non-linear model simple, tractable and interpretable. Given the nature of brain processes, we expect the true relationship between the high-dimensional fMRI signal and pain ratings to be a complex non-linear one. However, fitting an ad hoc non-linear model (e.g., a neural network) to such high-dimensional data to predict pain rating directly could be computationally much more challenging than fitting a linear one. On the other hand, given an accurate analytical model linking temperature to pain, we may exploit it advantages in our combined nonlinear method, first obtaining an estimate of the temperature from fMRI data via simple and computationally efficient linear regression, and then using nonlinear model predicting pain from temperature. Though the combined predictive model involves inferring temperature as a hidden variable, it outperforms the direct EN model because it captures (at least the temperature-to-pain part of) the non-linear relationship between fMRI and pain perception. To some extent, we can consider the analytic model as a principled constraint in the temporal domain, similar to the spatial regularization imposed by EN.

### Caveats, Limitations and Outlook

Our model can only provide a limited description of the full complexity of pain perception. In particular, the model accurately captures the perceptual dynamics in the time scale of seconds to minutes, most relevant for the functional interpretation of thermal pain as an “alarm signal”. Processes whose dynamics develop over longer time scales, such as habituation, sensitization, post-tissue injury, or following acute or chronic pain conditions [8], [11] are beyond the model's descriptive capabilities. For instance, repeated testing of offset analgesia over multiple days in [16] results in sensitization changes, which however do not alter the quality of the responses. Nevertheless, our model can provide an analytic framework even in the context of these long-term adaptive processes, as it will be possible to study the effect of adaptation on the different parameters that control the short-term perceptual dynamics, for instance threshold and decay time-constant. Another class of perceptual behaviors that our model does not consider, unrelated to differences in time scale, are those derived from interactions between pain and cognitive and attention processes, which can significantly modulate the perception to objectively similar noxious stimuli [40]–[41].

Despite its limitations, the model provides a powerful tool with which peripheral and central mechanisms can be studied. As the model describes subjective reports of magnitude of pain, it may also generalize to magnitude perception across other sensory modalities. Moreover, as we have tentatively shown with the combined model of fMRI-based prediction, it should be possible to identify physiological processes associated with the proposed components of the perceptual dynamics, and so reduce the gap between phenomenology and theory.

## Supporting Information

Figure S1
**Individual subjects and group averaged pain ratings and corresponding models.** Pain rating are shown in blue for simple (first two columns) and complex stimulus (3^rd^ and 4^th^ columns), fitted (red) with first (columns 1 and 3) and second order models (columns 2 and 4), corresponding parameters (first-order model: 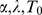; second-order model: 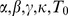) and fit correlations (*r*) are also presented. Stimulus temperature profiles are shown on top in green. **A.** Each row is a single subject. **B.** Group-averaged pain perception and calculated models. Note that group-averaged pain ratings for simple stimuli show better fit correlations than the individual subject models for the simple stimulus, and first-order and second-order models are essentially equivalent and show 97% similarity to the group-averaged pain rating. This is not the case for the second order model, due to its non-linear properties.(TIFF)

Figure S2 **A time expansion of part of** **fig. 2** **for the simple stimulus.** Stimulus and pain ratings are shown in balck, and first (top panel) and second (middle panel) order models are in red. The first order model consistently over estimates pain relief time profile, while the second order model captures this more accurately (compare corresponding arrows between top and bottom panels). Note that model performance measures do not capture such details as variability of rating within and across subjects dominates such measures.
(TIFF)

Figure S3 **Comparison between models and null hypotheses for fit correlations between the time derivative of the actual and modeled perception traces.** Panel A. comparison between the linear models and the 2nd order model, for simple and complex stimuli (circles and triangles, respectively). The linear model corresponds to the null hypothesis that perception follows temperature, while the threshold model assumes perception follows temperature above a threshold; the temperature threshold is optimized for each subject. But for a few cases (7 of 48), the second order model outperforms the linear and threshold models. Panel B. Same as Panel A, but the horizontal axis corresponds to the time derivative of the first order model. The first order model outperforms the second order model in 6 of 24 cases (this difference is statistically not significant, Wp, *p*>0.1). Data points correspond to values for each subject, and the dashed line to the identity.
(TIFF)

Figure S4 **Histograms of distributions of parameter values for all subjects and for simple and complex stimuli.** Panel A: distribution of the 3 parameters for the first order model. Panel B: distribution of the 5 parameters for the second order model.
(TIFF)

Figure S5
**Calculated threshold to pain and time constant are similar for first and second order models.** Left panel is the correlation of temperature threshold parameter (in degrees centigrade) between first and second order models, for simple and complex stimuli. Right panel is the time constant comparison between the two models (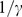 for the first order model, and 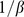 for the second order model) for the simple stimulus.(TIFF)

Figure S6 **Individual subjects perception and predicted perception based on parameters estimated from a previous pain rating.** Each row is an individual subject. Parameters estimated from pain rating run 1 are used to model perception for run 2 in each subject. Fit correlations are shown for each prediction. Column 1 is for simple stimulus using estimation from first order model (estimations are shown in Fig. S2, column 1); column 2 is the same data using estimations from second order model (column 2 in Fig. S2). Columns 3 and 4 are similar for the complex stimulus. Simple and complex stimuli are very well predicted for each subject by first and second order models.
(TIFF)

Figure S7 **Estimation and prediction relationship for individual subjects and group averages.** Panel A. The horizontal axis shows the fit correlation between pain perception and the best second order model for each subject (training, run 1, parameters estimated from this run); the vertical axis is the correlation between actual perception and predicted perception for a second independent pain rating (run 2), with the parameters learned from run 1 (test correlation). Open and full circles correspond to simple and complex stimuli conditions, respectively. As expected, test correlations, i.e. predictions, tend to be less accurate than training correlations. Panel B. Same as Panel A, but for parameters learned for the average response to the first run. In this case, predictions for complex stimuli are less accurate, as they reveal more clearly individual differences between the subjects. Panel C. Prediction of first order model for simple stimuli in run 2, based on estimates of complex stimuli on run1. Panel D. Prediction of second order model for complex stimuli in run 2, based on estimates for simple stimuli in run 1.Panel E. Prediction of second order model for simple stimuli in run 2, based on estimates for complex stimuli in run 1.
(TIFF)

Figure S8 **Rating intensity of burning pain or intensity of pain result in comparable models.** Panel A. Group average perception (*n* = 12 subjects) and predicted perception with corresponding estimated parameters for rating intensity of burning. Panel B. Group average for rating intensity of perceived pain (same as **Figure S1B**). Parameters and fit correlations are similar for both sets of instructions.
(TIFF)

Figure S9 **Comparison between the two-time-constant model and the second order model, for simple and complex stimuli.** Fit correlations were tested for equality, Wp, *p*>0.1, implying no difference between the two models.
(TIFF)

Figure S10 **Prediction of offset analgesia I.** We use group-averaged parameters (Figure S1) for the two models and apply the stimuli reported in figure 3 of Yelle et al. Panel A: patterns of temperature stimulation with different fall rates, from 0.5 to 5°C/sec. The 5 different patterns are color-coded. Panel B: result of simulating the first order model with group-averaged parameters and the stimulation patterns shown in Panel A. Panel C: same as Panel B, for the second order model.
(TIFF)

Figure S11 **Prediction of offset analgesia II.** Same as Figure S10, for stimuli reported in figure 1 of Derbyshire and Osborn. Panels A, D and G: temperature stimulation patterns. The colors indicate the plateau temperature reached. Panels B, E and H: result of simulating the first order model with the group-average parameters, for the corresponding stimulation patterns in the left column panels. Panels C, F and I: same as the center column panels, for the second order model.
(TIFF)

Figure S12
**Interpretation of the model I.** Panel A: a perception signal delayed with respect to the temperature may integrate the error to zero, while the 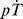
term is positive; conversely, an advanced perception signal will integrate the same term to a negative value. Panel B: the delayed signal implies a clockwise trajectory in the 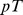 plane, leading to a positive integral for 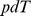; the converse is true for an advanced signal.(TIFF)

Figure S13
**Interpretation of the model II.** Panel A: Integration of the full Eq. 1 model (red trace), and a model without the 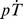
term (blue trace), for a temperature that consists of a mean above threshold and an oscillation on top of it (green trace). Panel B: Fourier analysis of the traces around the main frequency. The line traces correspond to the logarithm of the power, while the circles are the phases for each frequency.(TIFF)

Table S1 **Correlation between fitted parameters for the second- and first-order models.** Statistically significant correlations are indicated in bold-face.
(RTF)

Text S1 **Description of additional data, including fits to individual perception traces, and further comparisons between the proposed models.** An interpretation of the second order model in terms of signal processing is also included.
(DOCX)

## References

[1] Perl ER (2011) Pain mechanisms: a commentary on concepts and issues. Prog Neurobiol 94: 20. 21419824 10.1016/j.pneurobio.2011.03.001 PMC3138063

[2] Finger S (2001) Origins of Neuroscience: A history of explorations into brain function. London: Oxford University Press.

[3] Wittgenstein L (1953) Philosophical investigations. Oxford: Blackwell Publishing (English Translation 2001).

[4] Dennett DC (1978) Why you can't make a computer that feels pain. Synthese 38: 415.

[5] Merskey H, Bogduk N (1994) Classification of chronic pain. 2nd edition. Seattle: International Association for the Study of Pain.

[6] Weber EH (1978) The sense of touch. London: Academic Press.

[7] Stevens SS (1957) On the psychophysical law. Psychol Rev 64: 153. 13441853 10.1037/h0046162

[8] Price DD (1988), Psychological and neural mechanisms of pain. New York: Raven Press.

[9] Hansson P, Haanpaa M (2007) Diagnostic work-up of neuropathic pain: computing, using questionnaires or examining the patient? Eur J Pain 11: 367. 17276110 10.1016/j.ejpain.2006.12.005

[10] Cruccu G, et al (2004) EFNS guidelines on neuropathic pain assessment. Eur J Neurol 11: 153. 15009162 10.1111/j.1468-1331.2004.00791.x

[11] Hardy JD, Stolwijk AJ, Hoffman D, Kenshalo DR (1968) Pain following step increase in skin temperature. The skin senses. pp. 444–456. Springfield: Thomas.

[12] Ahlquist ML, Edwall LG, Franzen OG, Haegerstam GA (1984) Perception of pulpal pain as a function of intradental nerve activity. Pain 19: 353–366. 6483451 10.1016/0304-3959(84)90081-2

[13] Vierck CJ, Cooper BY, Franzen D, Ritz LA, Greenspan JD (1983) Behavioral analysis of CNS pathways and transmitter systems involved in conduction and inhibition of pain sensations and reactions in primates. In: Progress in Psychobiology. New York: Academic Press.

[14] Baliki MN, Geha PY, Apkarian AV (2009) Parsing pain perception between nociceptive representation and magnitude estimation. J Neurophysiol 101: 875–887. 19073802 10.1152/jn.91100.2008 PMC3815214

[15] Handwerker H, Schoedel A, Zimmermann K, Forster C (2005) How do pain ratings change the brain activation by painful stimuli measured with fMRI? Society for Neuroscience Abstracts

[16] Yelle MD, Rogers JM, Coghill RC (2008) Offset analgesia: a temporal contrast mechanism for nociceptive information. Pain 134: 174–186. 17533118 10.1016/j.pain.2007.04.014 PMC2213795

[17] Davis KD, Pope GE, Crawley AP, Mikulis DJ (2002) Neural correlates of prickle sensation: a percept-related fMRI study. Nat Neurosci 5: 1121–1122. 12368810 10.1038/nn955

[18] Davis KD, Pope GE, Crawley AP, Mikulis DJ (2004) Perceptual illusion of “paradoxical heat” engages the insular cortex. J Neurophysiol 92: 1248–1251. 15277602 10.1152/jn.00084.2004

[19] Yelle MD, Oshiro Y, Kraft RA, Coghill RC (2009) Temporal filtering of nociceptive information by dynamic activation of endogenous pain modulatory systems. J Neurosci 29: 10264–10271. 19692600 10.1523/JNEUROSCI.4648-08.2009 PMC2739444

[20] Amris K, Waehren EE, Jespersen A, Bliddal H, Danneskiold-Samsoe B (2011) Observation-based assessment of functional ability in patients with chronic widespread pain: A cross-sectional study. Pain 152: 2470–2476. 21715094 10.1016/j.pain.2011.05.027

[21] Morley S (2008) Psychology of pain. Br J Anaesth 101: 25–31. 18511440 10.1093/bja/aen123

[22] Mitchell TM, et al (2008) Predicting human brain activity associated with the meanings of nouns. Science 320: 1191–1195. 18511683 10.1126/science.1152876

[23] Kay KN, Naselaris T, Prenger RJ, Gallant JL (2008) Identifying natural images from human brain activity. Nature 452: 352–355. 18322462 10.1038/nature06713 PMC3556484

[24] Wager TD, Atlas LY, Leotti LA, Rilling JK (2011) Predicting individual differences in placebo analgesia: Contributions of brain activity during anticipation and pain experience. J Neurosci 31: 439–452. 21228154 10.1523/JNEUROSCI.3420-10.2011 PMC3735131

[25] Brown JE, Chatterjee N, Younger J, Mackey S (2011) Towards a physiology-based measure of pain: patterns of human brain activity distinguish painful from non-painful thermal stimulation. PLoS ONE 6: e24124. 21931652 10.1371/journal.pone.0024124 PMC3172232

[26] Carroll MK, Cecchi GA, Rish I, Garg R, Rao AR (2009) Prediction and interpretation of distributed neural activity with sparse models. Neuroimage 44: 112–122. 18793733 10.1016/j.neuroimage.2008.08.020

[27] Rish I, Cecchi GA, Baliki MN, Apkarian AV (2010) Sparse regression models of pain perception. Lect N Comp Sci 6334: 212–223.

[28] Hashmi JA, Davis KD (2008) Effect of static and dynamic heat pain stimulus profiles on the temporal dynamics and interdependence of pain qualities, intensity, and affect. J Neurophysiol 100: 1706–1715. 18701756 10.1152/jn.90500.2008

[29] Basbaum AI, Bautista DM, Scherrer G, Julius D (2009) Cellular and molecular mechanisms of pain. Cell 139: 267–284. 19837031 10.1016/j.cell.2009.09.028 PMC2852643

[30] Derbyshire SWG, Osborn J (2008) Enhancement of offset analgesia during sequential testing. Eur J Pain 12: 980–989. 18321740 10.1016/j.ejpain.2008.01.008

[31] Burnham KP, Anderson DR (2002) Model selection and multimodel inference: A practical information-theoretic approach. New York: Springer-Verlag.

[32] Zou H, Hastie T (2005) Regularization and variable selection via the Elastic Net. J Royal Stat Soc B 67: 301–320.

[33] Tibshirani R (1996) Regression shrinkage and selection via the lasso. J Royal Stat Soc B 58: 267–288.

[34] Hoerl A, Kennard R (1988) Ridge regression. Encyclopedia of Statistical Sciences 8: 129–136.

[35] Fu W (1998) Penalized regression: the bridge versus the lasso. J Comput Graph Statist 7: 397–416.

[36] Phillips JR, Johnson KO, Hsiao SS (1988) Spatial pattern representation and transformation in monkey somatosensory cortex. Proc Natl Acad Sci U S A 85: 1317–1321. 3422492 10.1073/pnas.85.4.1317 PMC279758

[37] Romo R, Salinas E (2001) Touch and go: decision-making mechanisms in somatosensation,. Annu Rev Neurosci 24: 107–137. 11283307 10.1146/annurev.neuro.24.1.107

[38] Gabernet L, Jadhav SP, Feldman DF, Carandini M, Scanziani M (2005) Somatosensory integration controlled by dynamic thalamocortical feed-forward inhibition. Neuron 48: 315–327. 16242411 10.1016/j.neuron.2005.09.022

[39] Dan Y, Atick JJ, Reid RC (1996) Efficient coding of natural scenes in the lateral geniculate nucleus: experimental test of a computational theory. J Neurosci 16: 3351–3362. 8627371 10.1523/JNEUROSCI.16-10-03351.1996 PMC6579125

[40] Miron D, Duncan GH, Bushnell MC (1989) Effects of attention on the intensity and unpleasantness of thermal pain. Pain 39: 345–352. 2616184 10.1016/0304-3959(89)90048-1

[41] Bantick SJ, Wise RG, Ploghaus A, Clare S, Smith SM, Tracey I (2002) Imaging how attention modulates pain in humans using functional MRI. Brain 125: 310–319. 11844731 10.1093/brain/awf022

