# Supplementary figures and images for: Predictive Dynamics of Human Pain Perception
Source: PLoS Comput Biol. 2012 Oct 25;8(10):e1002719. doi: 10.1371/journal.pcbi.1002719 (PMC3486880; doi:10.1371/journal.pcbi.1002719)

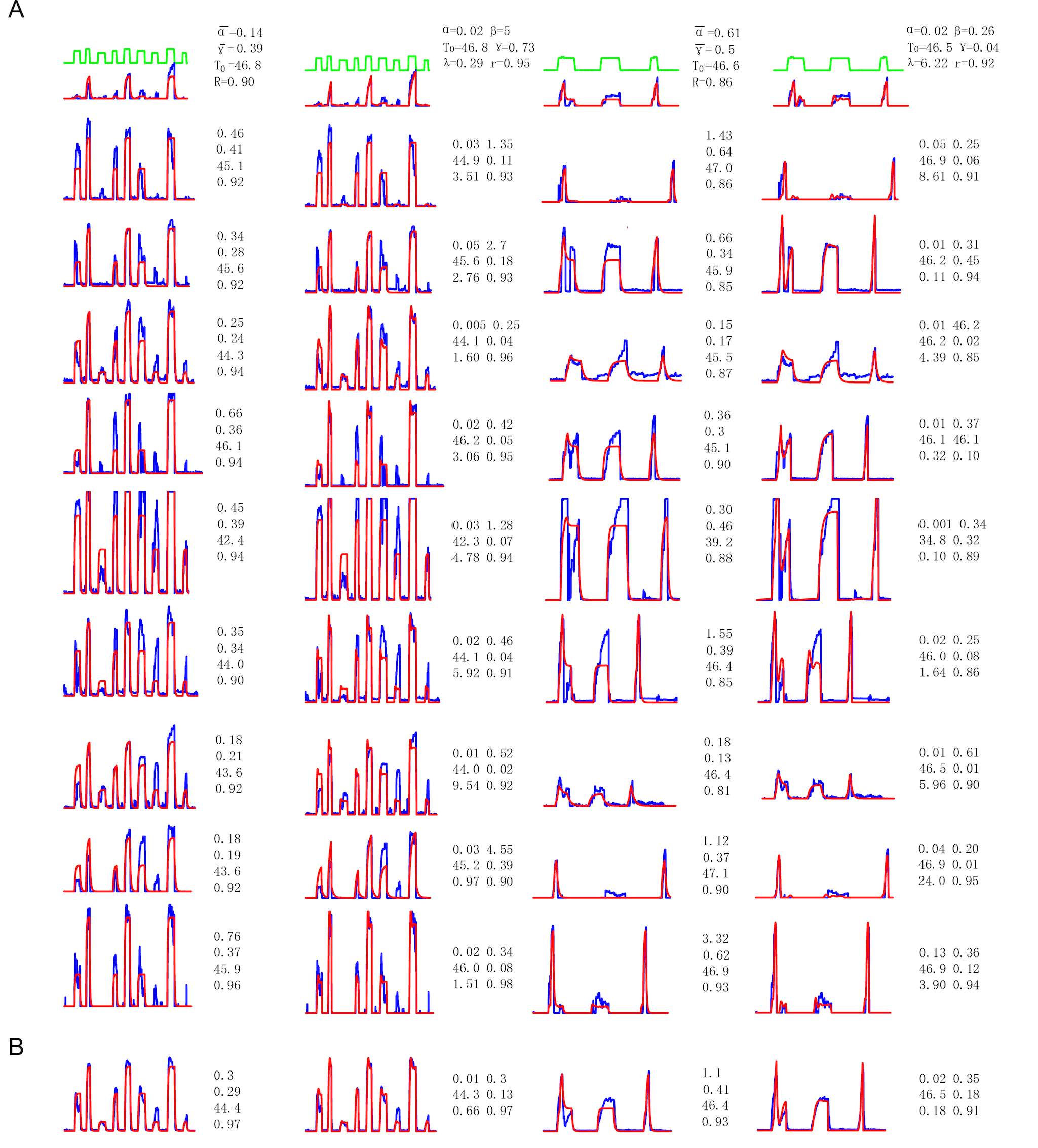

Supplement: Figure S1 — Individual subjects and group averaged pain ratings and corresponding models. Pain rating are shown in blue for simple (first two columns) and complex stimulus (3rd and 4th columns), fitted (red) with first (columns 1 and 3) and second order models (columns 2 and 4), corresponding parameters (first-order model: ; second-order model: ) and fit correlations (r) are also presented. Stimulus temperature profiles are shown on top in green. A. Each row is a single subject. B. Group-averaged pain perception and calculated models. Note that group-averaged pain ratings for simple stimuli show better fit correlations than the individual subject models for the simple stimulus, and first-order and second-order models are essentially equivalent and show 97% similarity to the group-averaged pain rating. This is not the case for the second order model, due to its non-linear properties. (TIFF) [file pcbi.1002719.s001.tiff]

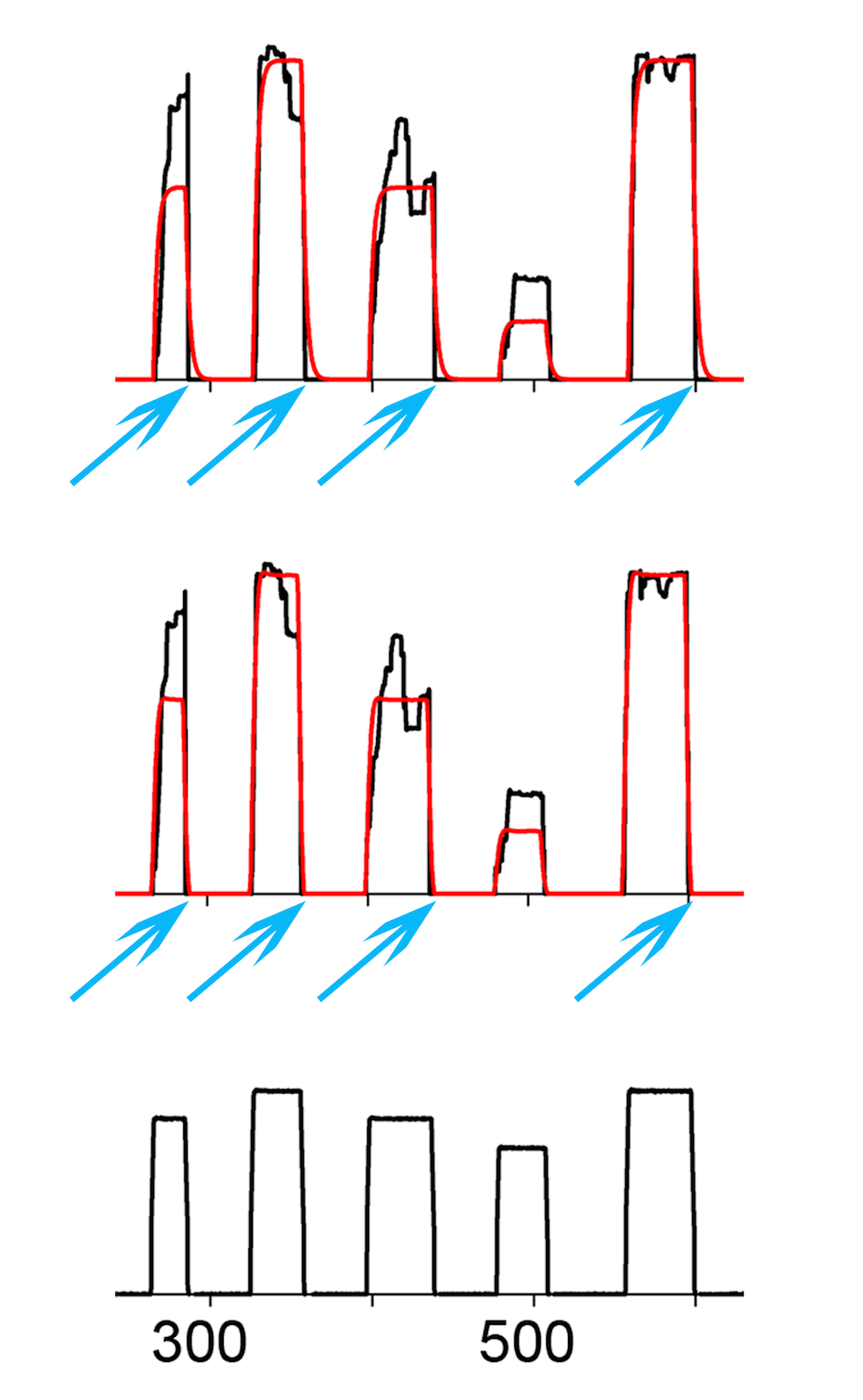

Supplement: Figure S2 — A time expansion of part of fig. 2 for the simple stimulus. Stimulus and pain ratings are shown in balck, and first (top panel) and second (middle panel) order models are in red. The first order model consistently over estimates pain relief time profile, while the second order model captures this more accurately (compare corresponding arrows between top and bottom panels). Note that model performance measures do not capture such details as variability of rating within and across subjects dominates such measures. (TIFF) [file pcbi.1002719.s002.tiff]

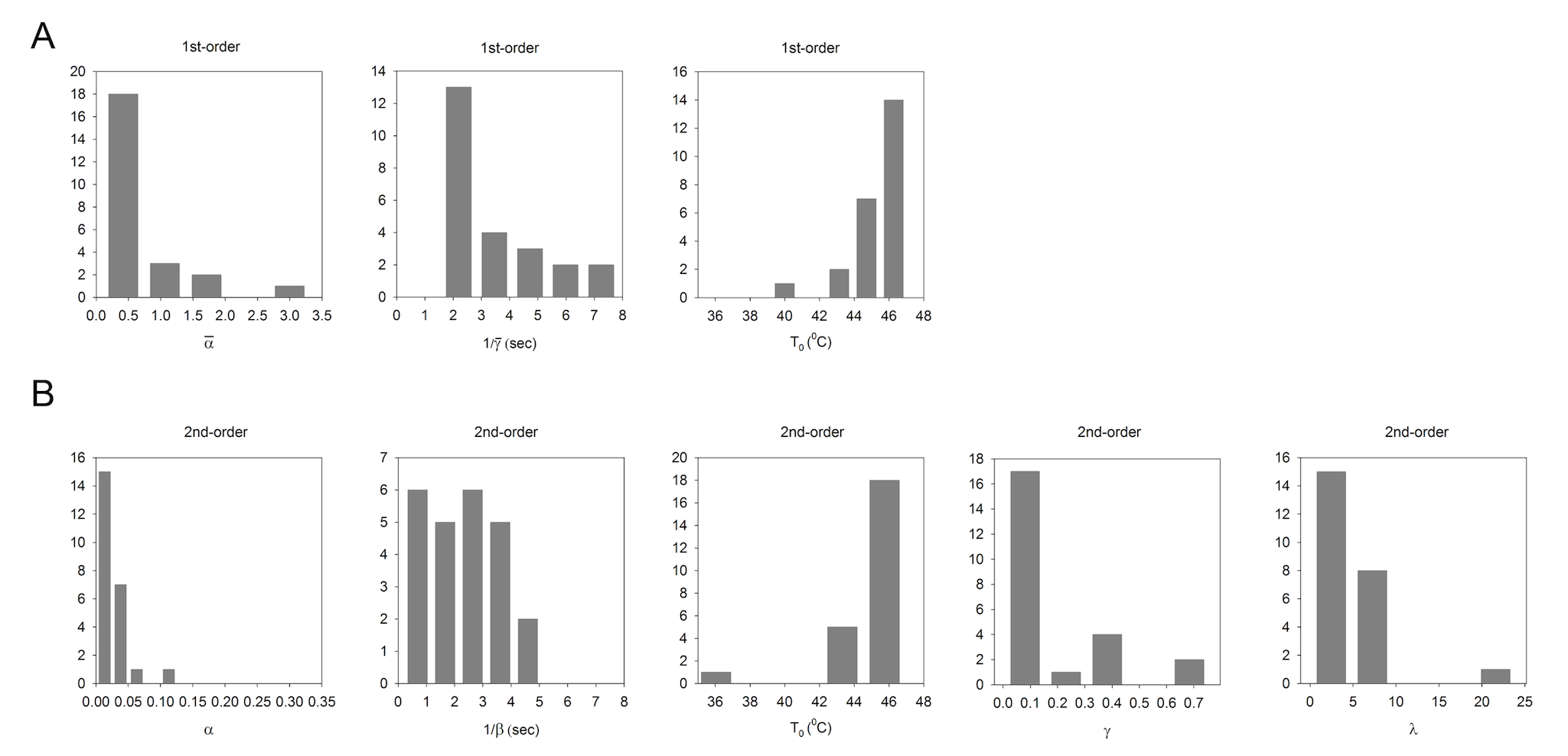

Supplement: Figure S4 — Histograms of distributions of parameter values for all subjects and for simple and complex stimuli. Panel A: distribution of the 3 parameters for the first order model. Panel B: distribution of the 5 parameters for the second order model. (TIFF) [file pcbi.1002719.s004.tiff]

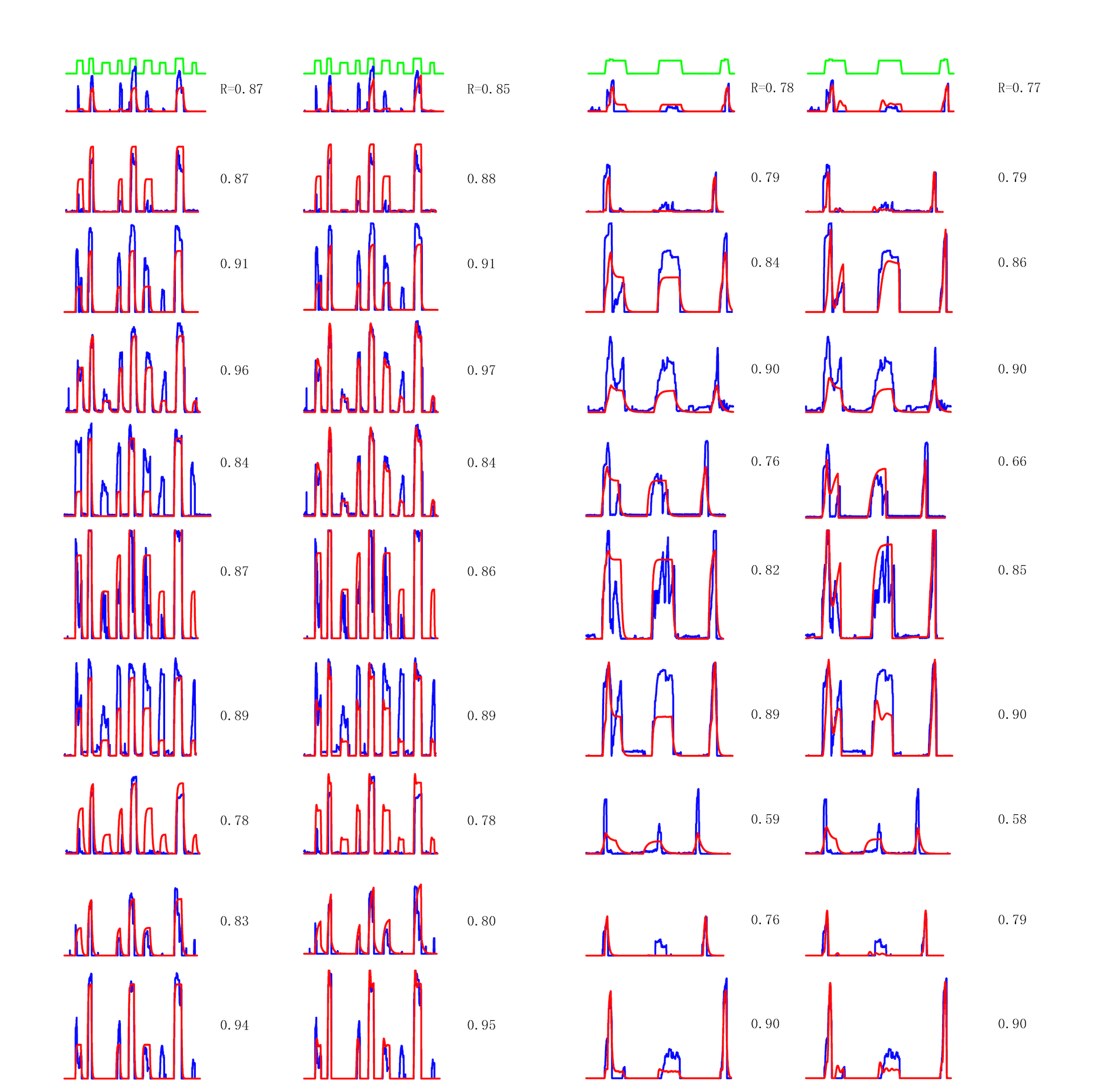

Supplement: Figure S6 — Individual subjects perception and predicted perception based on parameters estimated from a previous pain rating. Each row is an individual subject. Parameters estimated from pain rating run 1 are used to model perception for run 2 in each subject. Fit correlations are shown for each prediction. Column 1 is for simple stimulus using estimation from first order model (estimations are shown in Fig. S2, column 1); column 2 is the same data using estimations from second order model (column 2 in Fig. S2). Columns 3 and 4 are similar for the complex stimulus. Simple and complex stimuli are very well predicted for each subject by first and second order models. (TIFF) [file pcbi.1002719.s006.tiff]

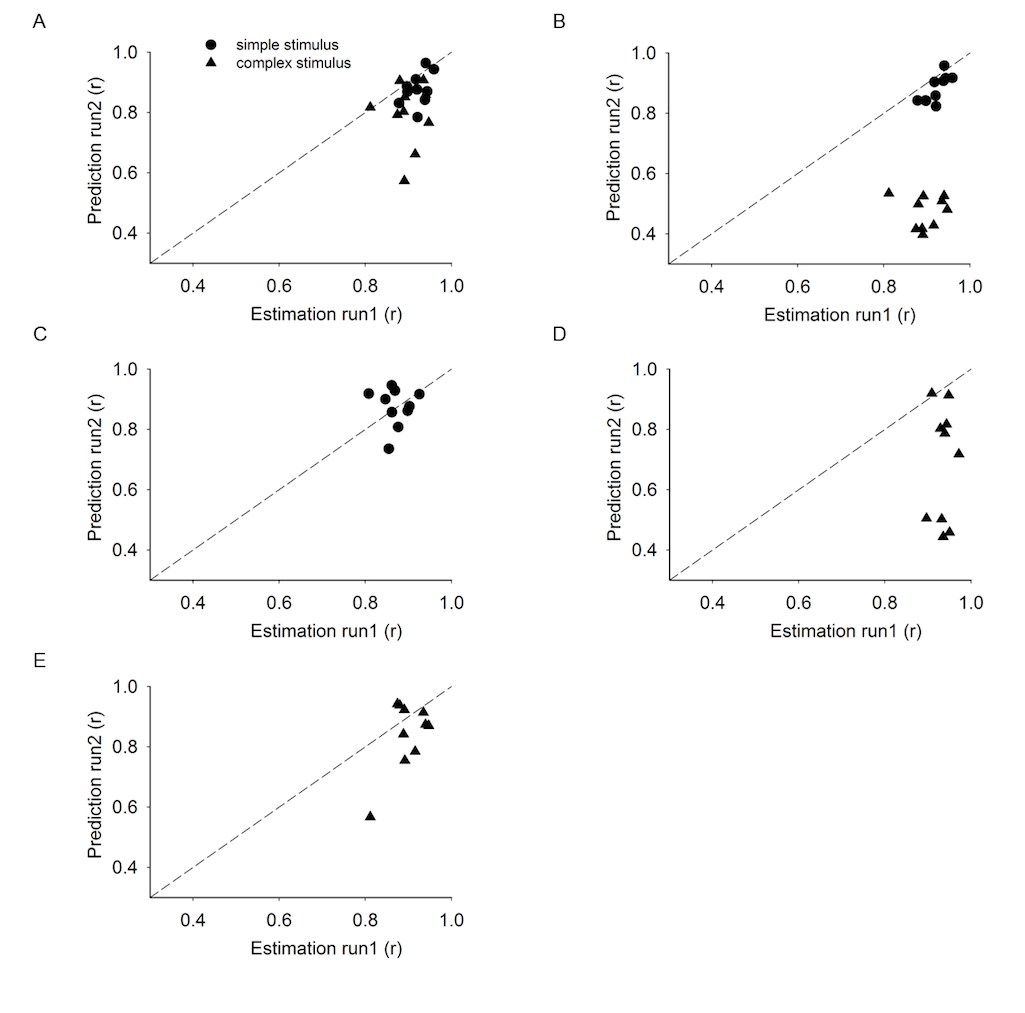

Supplement: Figure S7 — Estimation and prediction relationship for individual subjects and group averages. Panel A. The horizontal axis shows the fit correlation between pain perception and the best second order model for each subject (training, run 1, parameters estimated from this run); the vertical axis is the correlation between actual perception and predicted perception for a second independent pain rating (run 2), with the parameters learned from run 1 (test correlation). Open and full circles correspond to simple and complex stimuli conditions, respectively. As expected, test correlations, i.e. predictions, tend to be less accurate than training correlations. Panel B. Same as Panel A, but for parameters learned for the average response to the first run. In this case, predictions for complex stimuli are less accurate, as they reveal more clearly individual differences between the subjects. Panel C. Prediction of first order model for simple stimuli in run 2, based on estimates of complex stimuli on run1. Panel D. Prediction of second order model for complex stimuli in run 2, based on estimates for simple stimuli in run 1.Panel E. Prediction of second order model for simple stimuli in run 2, based on estimates for complex stimuli in run 1. (TIFF) [file pcbi.1002719.s007.tiff]

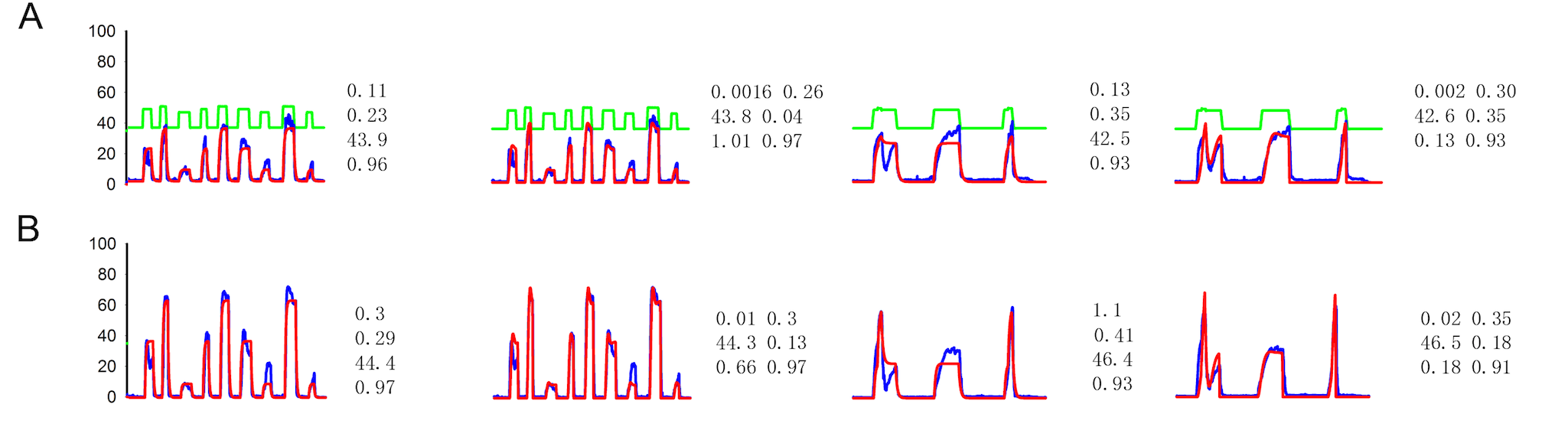

Supplement: Figure S8 — Rating intensity of burning pain or intensity of pain result in comparable models. Panel A. Group average perception (n = 12 subjects) and predicted perception with corresponding estimated parameters for rating intensity of burning. Panel B. Group average for rating intensity of perceived pain (same as Figure S1B). Parameters and fit correlations are similar for both sets of instructions. (TIFF) [file pcbi.1002719.s008.tiff]

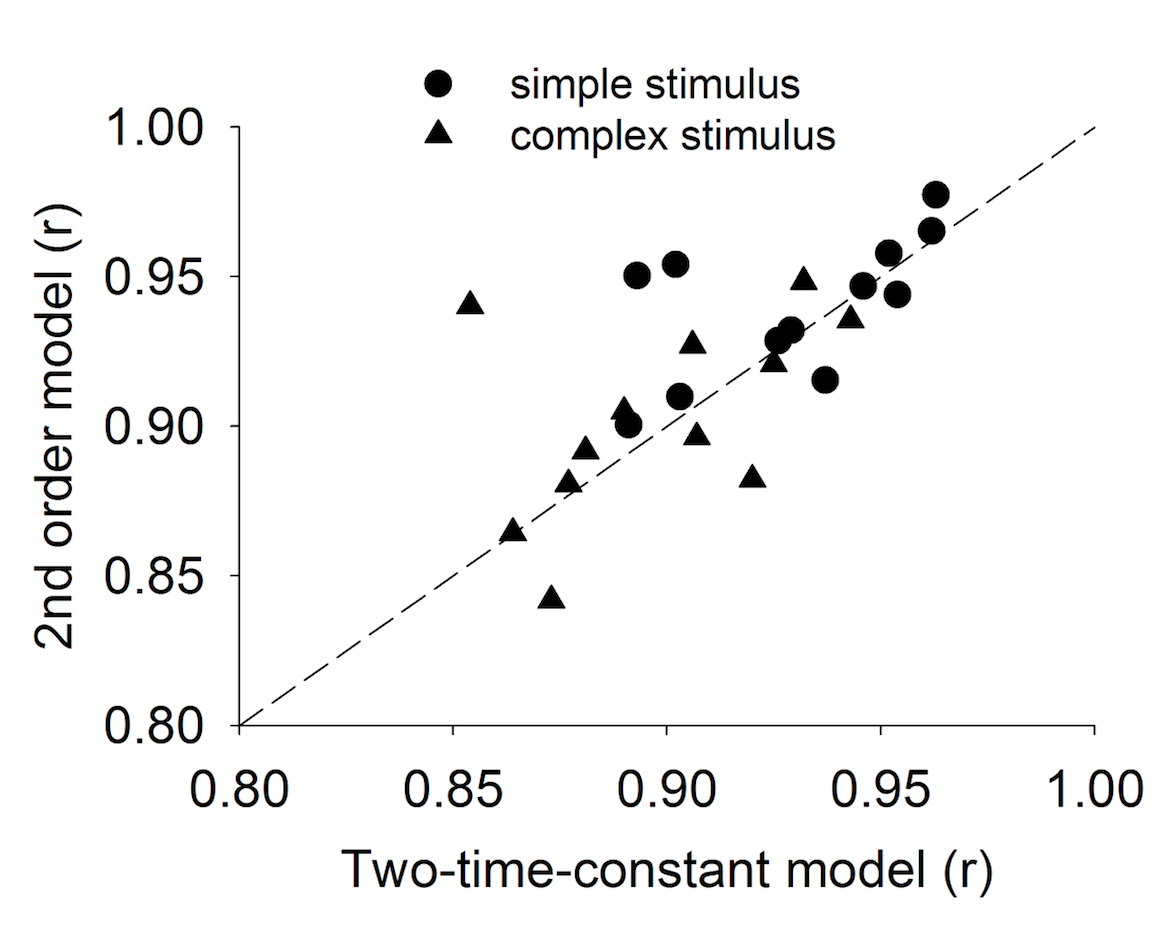

Supplement: Figure S9 — Comparison between the two-time-constant model and the second order model, for simple and complex stimuli. Fit correlations were tested for equality, Wp, p>0.1, implying no difference between the two models. (TIFF) [file pcbi.1002719.s009.tiff]

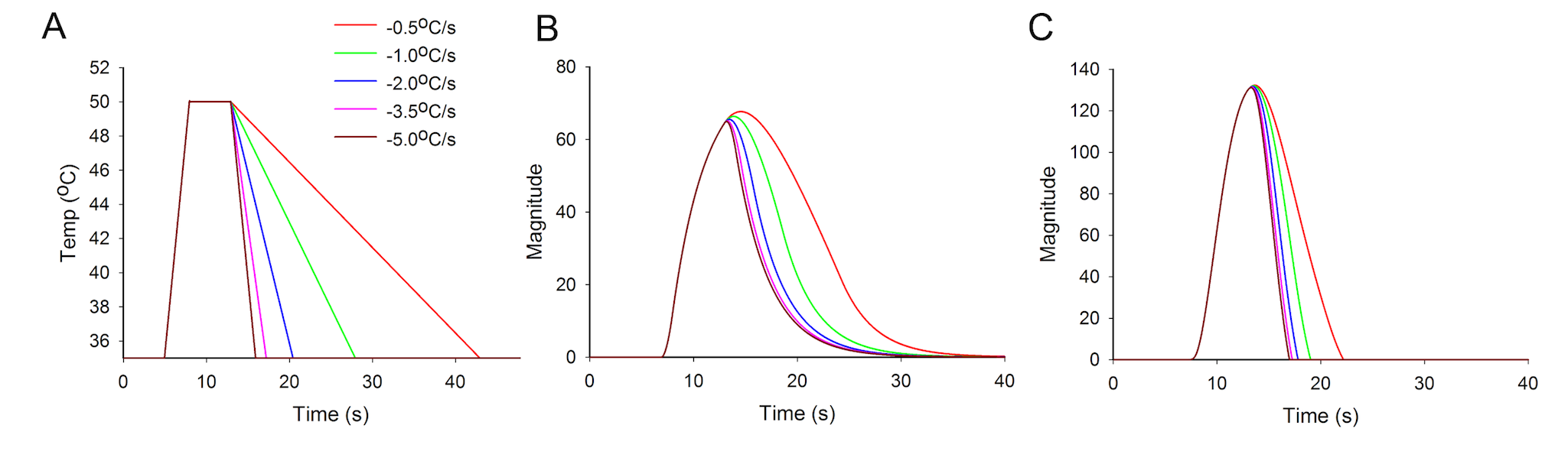

Supplement: Figure S10 — Prediction of offset analgesia I. We use group-averaged parameters (Figure S1) for the two models and apply the stimuli reported in figure 3 of Yelle et al. Panel A: patterns of temperature stimulation with different fall rates, from 0.5 to 5°C/sec. The 5 different patterns are color-coded. Panel B: result of simulating the first order model with group-averaged parameters and the stimulation patterns shown in Panel A. Panel C: same as Panel B, for the second order model. (TIFF) [file pcbi.1002719.s010.tiff]

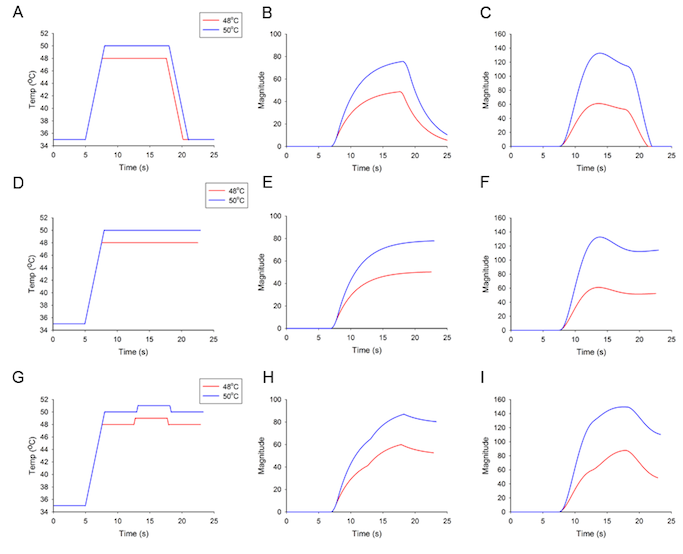

Supplement: Figure S11 — Prediction of offset analgesia II. Same as Figure S10, for stimuli reported in figure 1 of Derbyshire and Osborn. Panels A, D and G: temperature stimulation patterns. The colors indicate the plateau temperature reached. Panels B, E and H: result of simulating the first order model with the group-average parameters, for the corresponding stimulation patterns in the left column panels. Panels C, F and I: same as the center column panels, for the second order model. (TIFF) [file pcbi.1002719.s011.tiff]

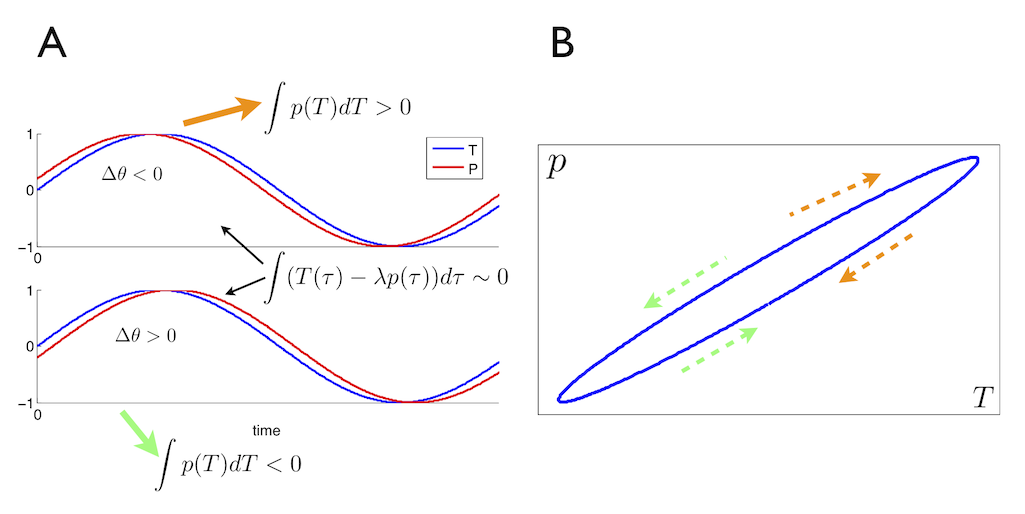

Supplement: Figure S12 — Interpretation of the model I. Panel A: a perception signal delayed with respect to the temperature may integrate the error to zero, while the term is positive; conversely, an advanced perception signal will integrate the same term to a negative value. Panel B: the delayed signal implies a clockwise trajectory in the plane, leading to a positive integral for ; the converse is true for an advanced signal. (TIFF) [file pcbi.1002719.s012.tiff]

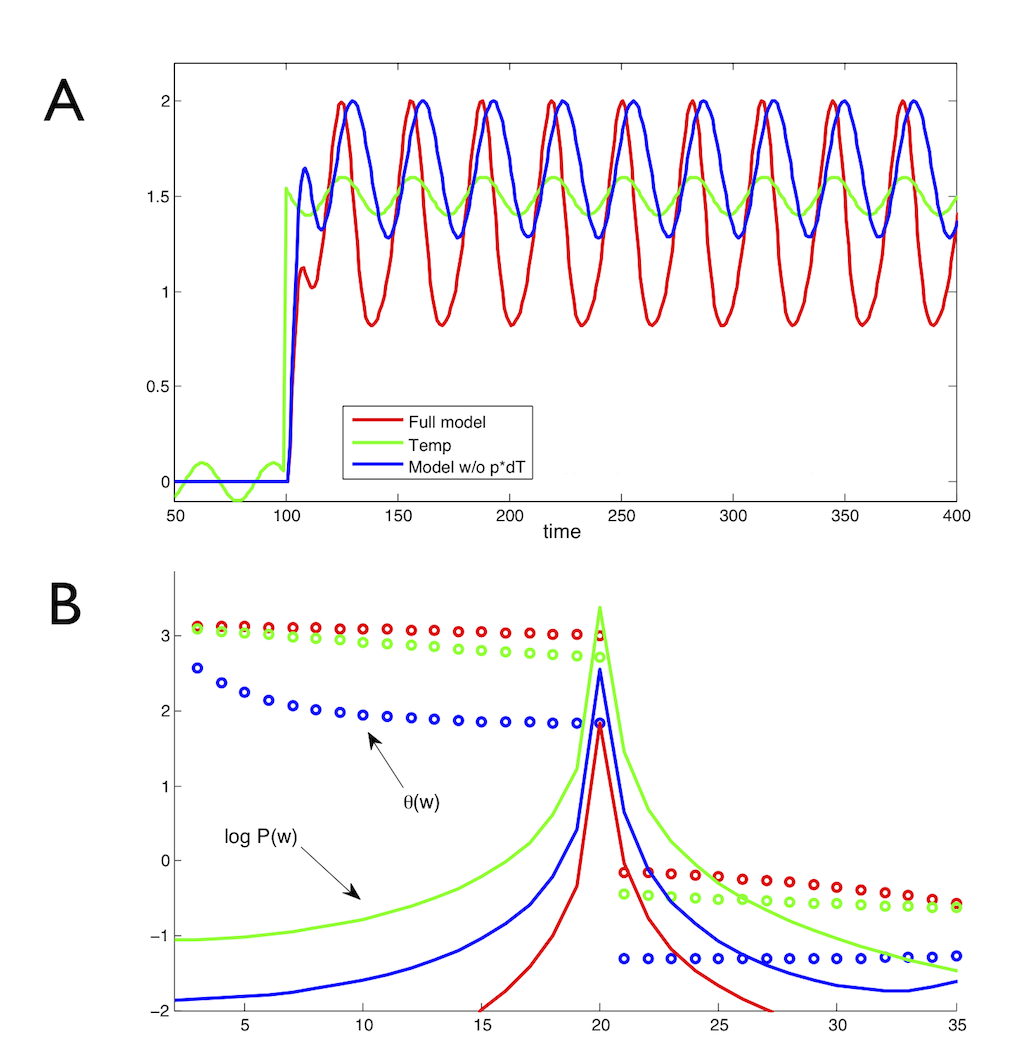

Supplement: Figure S13 — Interpretation of the model II. Panel A: Integration of the full Eq. 1 model (red trace), and a model without the term (blue trace), for a temperature that consists of a mean above threshold and an oscillation on top of it (green trace). Panel B: Fourier analysis of the traces around the main frequency. The line traces correspond to the logarithm of the power, while the circles are the phases for each frequency. (TIFF) [file pcbi.1002719.s013.tiff]
