# Supplementary material for: Predictive Dynamics of Human Pain Perception
Source: PLoS Comput Biol. 2012 Oct 25;8(10):e1002719. doi: 10.1371/journal.pcbi.1002719 (PMC3486880; doi:10.1371/journal.pcbi.1002719)
Supplement: Table S1 — Correlation between fitted parameters for the second- and first-order models. Statistically significant correlations are indicated in bold-face. (RTF) [file pcbi.1002719.s014.rtf]

Second Order Model
parameters	correlation	p-value	
,	0.56	0.01	
,	-0.53	0.017	
,	0.03	0.89	
,	-0.22	0.34	
,	0.14	0.55	
,	-0.34	0.14	
,	0.34	0.14	
,	0.03	0.9	
,	-0.10	0.66	
,	0.28	0.22	

First Order Model
parameters	correlation	p-value	
,	0.72	0.0003	
,	0.39	0.089	
,	0.06	0.80	
