# Supplementary material for: Predictive Dynamics of Human Pain Perception
Source: PLoS Comput Biol. 2012 Oct 25;8(10):e1002719. doi: 10.1371/journal.pcbi.1002719 (PMC3486880; doi:10.1371/journal.pcbi.1002719)
Supplement: Text S1 — Description of additional data, including fits to individual perception traces, and further comparisons between the proposed models. An interpretation of the second order model in terms of signal processing is also included. (DOCX) [file pcbi.1002719.s015.docx]

**Comparing pain perception predictions with first and second order models**

We tested our equations by collecting continuous pain ratings in healthy subjects in response to a simple stimulus and a complex stimulus. We speculated that the second order equation will be more effective in capturing pain responses evoked by the complex stimulus that has been shown in previous studies to evoke a disproportional nonlinear drop in pain following a small decrease in supra-threshold temperatures, a phenomenon labeled as offset analgesia. **Figure 1** and **Figure 2** show that the temporal properties of perceived pain for the complex stimulus is better captured for published results and in a given subject in our psychophysical results. All subjects in our study reported a comparable offset analgesia in response to the small decrease in temperature and this nonlinear decrease in pain was captured closely by the second order equation. In contrast, the first order equation was similarly effective as the second order equation in correlating with the pain time course evoked by the simple stimulus (**Figure S1**).

**Figure S2** is an expanded version of part of **Figure 2**, for the simple stimulus in one subject. The figure illustrates that even for the simple stimulus the second order model better captures the time characteristics of pain relief.

Correlations (zero lag) between pain ratings and calculated models (based on parameters identified by fitting each model ratings, as shown for each case in **Figure S1**) were used to assess performance. **Figures 2G, H** show that fit correlations are larger for the second order model in contrast to null hypotheses and in contrast to first order model for both simple and complex stimuli. Wilcoxon matched-pairs signed-ranks test, Wp, between first order model and second order model fit correlations for both simple and complex stimuli *p*<0.02.

We also compared the correlation between the derivatives of the rating and of the model, a measure that was not directly optimized for the fit (**Figure S3**). While there are more cases in which the second-order model underperforms in comparison with the null hypotheses and the simplified first-order model, it is still evident that it largely captures much better the more detailed temporal information carried by the derivative of the rating. Distributions of fit parameters for first and second order models, for all subjects, are shown in **Figure S4**. Equivalent parameters show similar distributions between the two models. The parameter that directly relates between the two models is threshold and its values closely match between the two models (**Figure S5**). In the second order model, the time-constant can be approximated by **$1/\beta$**, and this measure does not differ from the first order model time-constant (**Figure S5**). Calculated thresholds and time constants are similar to results reported in the literature.

We next tested the extent of generalization of obtained results by using part of the data as a training data set and the remaining for predictions based on model parameters calculated from the training. Individual subject pain rating parameters for simple and complex stimuli, and using first or second order models showed very high predictability (**Figure S6**). For simple stimuli parameter estimation fit correlation was 0.92± 0.01 (mean±SEM); while for prediction corresponding value was 0.88± 0.02, testing for equality of outcomes, using Wp, results in *p*<0.02, indicating that estimation is superior to prediction. Similarly, for the complex stimulus and using the second order model estimation fit correlation was 0.90±0.01; while for prediction corresponding value was 0.80±0.03, and these were not different from each other, Wp equality test *p*>0.1 (**Figure S7A**). Thus, individual subject pain ratings can be predicted adequately for simple and complex stimuli and for both models. Additionally we tested group-averaged parameters prediction of individual subjects pain ratings: For the simple stimulus and using first order model, for estimation we obtain a fit correlation of 0.92±0.01 and for prediction a fit correlation of 0.88±0.01, where estimation outcome was slightly better than prediction (Wp equality test, *p*<0.02). For the complex stimulus and using second order model, for estimation we obtain a fit correlation of 0.90±0.01 and for prediction a fit correlation of 0.47±0.02, where prediction outcome was much worse than estimation (Wp equality test, *p*>0.002) (**Figure S7B**). Thus, group average model parameters are adequate for simple stimuli and first order modeling, but fail for complex stimuli using second order modeling.

To test whether these models are applicable to other dimensions of pain, we collected ratings where subjects were instructed to specifically rate the magnitude of perceived dull burning. Group-averaged ratings and resultant models show close approximation to values we observe for rating pain intensity, even though the overall magnitude perception is lower for burning (**Figure S8**). We also tested an additional model where two separate time constants were used to account for myelinated and unmyelinated nociceptors. Resultant model did not perform better than the second order model (**Figure S9**).

Offset analgesia: As our second order model was designed to capture offset analgesia properties, we tested whether the model will also show less sensitivity to stimulus offset rates, in comparison to the first order model where perception fall rates should better reflect stimulus fall rates. **Figure S10** shows that in fact these predictions are correct: the model closely captures pain ratings as described in figures 3 and 4 of Yelle et al. 2008, using the average of the parameters in Fig. S1. The figure shows that stimulation with very different fall rates (**Figure S1-A**) results in perception fall rates for the second order model (**Figure S1-C**) that match better the experimental results than the first order model (**Figure S1-B**). A second observation is that pain perception magnitude for increasing intensities shows different patterns when the stimulus has an additional one degree perturbation (offset stimulus) in contrast to when the stimulus is kept at a constant level or returns to baseline (Derbyshire SW, Osborn J., Europ J Pain 2008). Again our second order model captures these features better than the first order, and in fact our model replicates figures 2-5 of Derbyshire and Osborn. This is shown in **Fig. S11**, which displays the stimulation patterns in **Figures S11-A**, **D** and **G**, the results for the first order model in **Figures S11-B**, **E** and **H**, and for the second order model in **Figures S11-C**, **F** and **I**. The authors also show that with repeated testing over multiple days these differences are further enhanced; while our model does not have such long-term adaptive parameters, as simple modification could account for this feature.

**Interpreting the model**

We can gain further insight into the behavior of the system described by Eq. 1. Integrating over time both sides of the equation leads, after rearranging terms, to:

**$\dot{p}(t)=\int\left(\alpha F(T(\tau),T_{0})-\lambda p(\tau)\right)d\tau-\beta p(t)+\gamma\int\dot{T}(\tau)p(\tau)d\tau$** (1)

The first term of the r.h.s. can be interpreted as running average of the error between temperature (above threshold) and perception, and the second term a decay factor to forget errors made in the distant past. More interesting is the last term, which can be written as an integral on the temperature, leading to:

**$\dot{p}(t)=\varepsilon(T)-\beta p(t)+\gamma\int p(T)dT$** (2)

This shows that the change in perception is driven by the mismatch between temperature and perception, and by a term that takes into consideration phase differences. In order to see this, we show in **Fig. S12** - Panel A, a toy example where the perception signal is delayed with respect to the temperature (upper graph) and another where it is advanced (lower graph). Panel B shows the plot of$p$ as a function of$T$, which makes clear that the delayed perception integrates the phase term to a positive value, in turn advancing perception to catch up with the temperature.

We present in **Figure S13** the result of integrating Eq. 1, i.e. the full model, and the same equation without the **$p\dot{T}$** term, for an input temperature that consists of an oscillation on top of a mean above the threshold. Panel A shows the resulting evolution of perception in both cases, making evident that the full model follows temperature more closely. In order to quantify this statement, we present in Panel B the distribution of phases of the Fourier components around the main frequency for the temperature and the two perception signals. This analysis shows that the phases of the full model follow the temperature much closer than the reduced model.
